# Genome analysis of a halophilic *Virgibacillus halodenitrificans* ASH15 revealed salt adaptation, plant growth promotion, and isoprenoid biosynthetic machinery

**DOI:** 10.3389/fmicb.2023.1229955

**Published:** 2023-09-22

**Authors:** Anjney Sharma, Ram Nageena Singh, Xiu-Peng Song, Rajesh Kumar Singh, Dao-Jun Guo, Pratiksha Singh, Krishan K. Verma, Yang-Rui Li

**Affiliations:** ^1^Key Laboratory of Sugarcane Biotechnology and Genetic Improvement, Ministry of Agriculture, Sugarcane Research Center, Chinese Academy of Agricultural Sciences, Guangxi Academy of Agricultural Sciences (GXXAS), Nanning, Guangxi, China; ^2^Guangxi Key Laboratory of Sugarcane Genetic Improvement, Sugarcane Research Institute, Guangxi Academy of Agricultural Sciences, Nanning, Guangxi, China; ^3^Department of Chemical and Biological Engineering, South Dakota School of Mines and Technology, Rapid City, SD, United States; ^4^State Key Laboratory of Conservation and Utilization of Subtropical, College of Agriculture, Agro-Bioresources, Guangxi University, Nanning, Guangxi, China

**Keywords:** *Virgibacillus halodenitrificans*, whole genome, salt-tolerant, plant growth promoting traits, CRISPRs, isoprenoids, squalene

## Abstract

Globally, due to widespread dispersion, intraspecific diversity, and crucial ecological components of halophilic ecosystems, *halophilic* bacteria is considered one of the key models for ecological, adaptative, and biotechnological applications research in saline environments. With this aim, the present study was to enlighten the plant growth-promoting features and investigate the systematic genome of a halophilic bacteria, *Virgibacillus halodenitrificans* ASH15, through single-molecule real-time (SMRT) sequencing technology. Results showed that strain ASH15 could survive in high salinity up to 25% (w/v) NaCl concentration and express plant growth-promoting traits such as nitrogen fixation, plant growth hormones, and hydrolytic enzymes, which sustain salt stress. The results of pot experiment revealed that strain ASH15 significantly enhanced sugarcane plant growth (root shoot length and weight) under salt stress conditions. Moreover, the sequencing analysis of the strain ASH15 genome exhibited that this strain contained a circular chromosome of 3,832,903 bp with an average G+C content of 37.54%: 3721 predicted protein-coding sequences (CDSs), 24 rRNA genes, and 62 tRNA genes. Genome analysis revealed that the genes related to the synthesis and transport of compatible solutes (glycine, betaine, ectoine, hydroxyectoine, and glutamate) confirm salt stress as well as heavy metal resistance. Furthermore, functional annotation showed that the strain ASH15 encodes genes for root colonization, biofilm formation, phytohormone IAA production, nitrogen fixation, phosphate metabolism, and siderophore production, which are beneficial for plant growth promotion. Strain ASH15 also has a gene resistance to antibiotics and pathogens. In addition, analysis also revealed that the genome strain ASH15 has insertion sequences and CRISPRs, which suggest its ability to acquire new genes through horizontal gene transfer and acquire immunity to the attack of viruses. This work provides knowledge of the mechanism through which *V. halodenitrificans* ASH15 tolerates salt stress. Deep genome analysis, identified MVA pathway involved in biosynthesis of isoprenoids, more precisely “Squalene.” Squalene has various applications, such as an antioxidant, anti-cancer agent, anti-aging agent, hemopreventive agent, anti-bacterial agent, adjuvant for vaccines and drug carriers, and detoxifier. Our findings indicated that strain ASH15 has enormous potential in industries such as in agriculture, pharmaceuticals, cosmetics, and food.

## Introduction

With the ever-changing climate and global warming, rising sea level are a growing concern which increase soil salinity across the coastal areas, thereby globally imposing a detrimental effect on soil quality, decreasing land areas, and reducing agricultural crop production, resulting in an instable national economy (Corwin, 2021; Godde et al., 2021; Ullah et al., 2021). Salinity stress causes changes in various physiological and metabolic processes of the plant, ultimately inhibiting the quality and productivity of agriculturally important crops (Sharma et al., 2021). In the current scenario, with limited cultivated land resources, growing crops in saline soil may be a feasible opportunity (Xiaoqin et al., 2021). In light of the rising global need for food and agricultural production, scientists are looking for new, more eco-friendly, greener, and more sustainable alternatives pesticides and chemical fertilizers (Ullah et al., 2021). In this framework, the unusual halophilic bacteria thriving in saline environments have been a subject of study for the last few years due to their interesting physiological and metabolic adaptation properties to their extreme environmental conditions (Dutta and Bandopadhyay, 2022). These incredibly resilient microorganisms can thrive at 0% to saturation salt concentrations. The basic mechanisms of these extremophiles microbes for halo-adaptation and surviving in saline habitats are based on two strategies to regulate intracellular osmolarity and evade water loss (Zhou et al., 2017). The first is the “salt-in” approach, where KCl inorganic salt is stored to provide the cellular osmotic pressure and balance the external osmotic pressure. The other is known as the “salt-out” (compatible solutes) strategy, in which low-molecular-weight, highly soluble organic molecules are cumulated to ensure cellular osmotic balance without hindering crucial cellular processes even when they are occurring at high levels (Sharma et al., 2016). Extremely halophilic and anaerobic moderately halophilic bacteria adopted the “salt-in” strategy, while the majority of bacteria used the “salt-out” (compatible solutes) approach (Zhou et al., 2017). There are numerous reports of new halophilic bacteria and archaea species available from diverse hypersaline locations in various nations, primarily from USA, Australia, Korea, China, India, Thailand, Taiwan, Russia, France, Austria, Spain, Japan, Egypt, Iran, Indonesia, Mexico, the Philippines, Poland, and Romania (Naghoni et al., 2017; Corral et al., 2019; Reang et al., 2022).

The exploitation of new halophilic microorganisms is always of special importance and interest in the current era for the evaluation and development of new biomolecules with potential applications in agriculture and several industries. Halophilic microorganisms help in the improvement of soil structure, plant salt tolerance, and growth through various mechanisms such as phytohormones production (IAA and ABA), solubilizing the essential nutrients (P, K, and Zn, etc.), regulating the ethylene level, and inducing systemic resistance (ISR) against the harmful plant pathogens through the production of secondary metabolite/antimicrobial peptides (Arora et al., 2020; Masmoudi et al., 2023). Additionally, halophilic microorganisms mitigate the salt stress in plants by maintaining high K^+^/Na^+^ and Ca^2+^ and scavenging ROS by regulating the expression of antioxidant enzymes and stress-responsive genes (Sharma et al., 2021). In addition, low nutritional requirements, genetic pieces of machinery, and great metabolic versatility for adaptation to harsh environmental environments make halophilic microorganisms a promising candidate and a hope for new sources of enzymes, drug discovery, and other biological materials with applications in various human welfare fields (Vaidya et al., 2018). Several extremely halophilic and halotolerant bacteria, such as *Bacillus, Haloferax, Micrococcus, Salinibacter Halobacterium, Halobacillus, Virgibacillus*, and *Haloarcula*, were reported from various saline environments (Gupta et al., 2015; Satari et al., 2021). Also, the plant growth-promoting effectiveness of so many halophilic bacteria was investigated in seed germination and growth promotion of several agriculturally important crops such as rice (Abbas et al., 2019; Suriani et al., 2020), tomato, cotton, maize (Anbumalar and Ashokumar, 2016), and sugarcane (Sharma et al., 2021).

Among the various bacterial genera inhabiting various extreme environments, the morphologically, biochemically, and genetically diverse genus *Virgibacillus* is widely recognized as an important model group for agriculture and industrial applications (Sánchez-Porro et al., 2014; Fayez et al., 2022). A wide range of different species of *Virgibacillus* have been reported globally from various saline environments, such as seawater, marine sediments, lakes, soil, and fermented seafoods (Montriwong et al., 2012; Amziane et al., 2013; Xu et al., 2018; Mechri et al., 2019; Bhatt and Singh, 2022). To date, 39 validated and 381 non-validated species in the *Virgibacillus* genus have been published in the NCBI database (Chen et al., 2018), whereas six complete genomic sequences of *Virgibacillus* species are available in the NCBI database (2017) (Chen et al., 2018). *Virgibacillus halodenitrificans* is one of such bacteria whose potential is less explored (Lee et al., 2012; Kumaunang et al., 2019; Fayez et al., 2022). In 1989, the first report of a halophilic denitrifier, *Bacillus halodenitrificans*, from a solar saltern was reported by Denariaz et al. (1989). The isolates grew and survived well in various NaCl (0.35 to 4.25 M NaCl) supplement mediums, with optimum growth was considered between 0.5 and 1.35 M (3 to 8%) NaCl. Later on, in 2004, *Bacillus halodenitrificans* was transferred into the genus *Virgibacillus* as *Virgibacillus halodenitrificans* (Yoon et al., 2004). Although numerous halophiles have been thoroughly defined to date, *V. halodenitrificans* is still one of the least explored organisms in terms of the number of published research studies, strain characterizations, and whole genome sequence analysis (Lee et al., 2012). Thus, in order to explore its capabilities to be commercialized, it is required to understand its genetic structure and metabolic mechanisms involved in the biosynthesis of beneficial biomolecules.

With the advancement of powerful tools and “omics” approaches such as whole-genome sequencing analysis, deciphering new insights into halophilic microorganisms (Durán-Viseras et al., 2021; Lam et al., 2021). Also, in response to the extreme environments, the concomitant advances in genomics are disclosing uncountable encoding genes for understanding the adaptation strategies, physiological attributes, and metabolic features of the halophilic bacteria (Corral et al., 2019). This resulted in a plethora of genomic information that was mined for potential agricultural and industrial applications (Ziemert et al., 2016; Othoum et al., 2019; Passari et al., 2019). Draft genomes with inaccurate or incomplete genomic data and low completeness are not fully reliable for phylogenomics, genome structure, genome synteny, and pan-genomic investigations (Denton et al., 2014). Thus, to know more about physiological, metabolic, and functional mechanisms, there is a need to generate high-quality whole genome sequences of halophilic microorganisms on a large scale to further understand the complete role of genes and their proteins in various extreme environments. The information from whole genome sequence analysis will constitute an exciting period for microbiology and the allied sector in the near future, which is generally not fully explored in the draft genome sequencing analysis. Since the first report of *Virgibacillus halodenitrificans* (Denariaz et al., 1989; Yoon et al., 2004), only one draft genome (Lee et al., 2012) and one complete genome (Zhou et al., 2017) have been published.

Therefore, in this study, we isolated and characterized a halophilic bacterium *Virgibacillus halodenitrificans* strain ASH15, from sugarcane-grown field in the coastal regions of Beihai, China and sequenced its entire genome. In addition, the plant growth-promoting efficiency of *V. halodenitrificans* ASH15 was evaluated for sugarcane plant growth under greenhouse conditions. Furthermore, systematic analysis of whole genome sequence data and the identification of genes will aid our understanding of the molecular mechanisms of osmoadaptation and the metabolic activities of the strain. Moreover, the obtained genome information will help to develop the strain ASH15 as an eco-friendly model for industrially important biomolecules and sustainable agriculture production.

## Materials and methods

### Sampling and isolation of halophilic bacteria

The soil sample was collected from the sugarcane-growing field of the sea city of Beihai, China (Latitude 21.4811° N, Longitude 109.1201° E). For the isolation of specific halophilic bacteria, the collected soil sample was enriched in a nutrient broth (NB) medium containing 10% NaCl at 37°C for 72 h. After the enrichment, the enriched soil sample was heat-treated at 80°C for 15 minutes to kill vegetative cells (Sharma et al., 2015). Spore-forming halophilic bacteria were isolated through the standard serial dilution method by spreading the diluted soil sample (10^−4^) over a nutrient agar (NA) growth medium plate supplemented with 10% NaCl. Plates were incubated at 37°C for 72 h. After the incubation, a dominant bacterial isolate designated as ASH15 was recovered and purified by sub-culturing on the same NaCl-amended growth medium. The purified culture was preserved in 50% glycerol stock at −80°C for further study.

### Growth kinetics studies under different levels of NaCl stress

Bacterial growth kinetics under different NaCl concentrations was spectroscopically determined in a 96-well microplate at 37°C. In brief, 1% of pure bacterial culture was transferred to an individual well containing 200 μl of NB broth with different NaCl concentrations, *viz*., 0, 5, 10, 15, 20, and 25%, and incubated to grow at 37°C under shaking condition at 150 rpm. The growth was spectroscopically monitored in a microplate reader by taking absorbance at 600 nm at every 12 h time interval.

### Scanning electron microscopic analysis

A pure single bacterial colony was inoculated in NB and incubated for 48 h at 37°C under shaking conditions. After incubation, the cell pellet of bacterial culture was collected via centrifugation at 10,000 rpm at room temperature for 10 min. The collected pellet was washed 2–3 times with 100 mM phosphate buffer and then fixed with a 2.5% glutaraldehyde solution and incubated at 4°C for 10–12 h. After fixation, treated cells were further washed with phosphate buffer and with a gradient concentration of ethanol (10 to 100%) every 10 min. Following by, sample was dried in desiccators, mounted onto SEM stubs, and coated thinly with gold and palladium (60:40), and then sample was examined using the SEM machine.

### Metabolic characterization through BIOLOG

The metabolic potentiality of the strain ASH15 was tested based on the carbon (C) utilization pattern in the BIOLOG Micro-Array TM GENIII plate (Biolog Inc., Hayward, CA) which contained 95 different carbon sources. Briefly, a single pure colony of strain ASH15 was streaked on NA plates and incubated at 30°C for 24 h. After the incubation, the bacterial culture mass was scraped from the surface of the plate and transferred into a sterile 2-ml centrifuge tube. Collected cells were washed with phosphate buffer and suspended in 20 ml of inoculation fluid (IF) (Biolog Inc., Hayward, CA) to reach a transmittance of 81–85% as per the manufacturer's instructions. A 100 μl of suspension was inoculated in each well of the GNIII plate, which contained 95 different C sources. After incubation to record the results, the plate was read in an automated BIOLOG(R) Micro-Station Reader according to the manufacturer's instructions.

### Biofilm formation and motility assay

The biofilm formation capacity of the strain ASH15 was assayed according to the method of Qurashi and Sabri (2012). 100 μl of pure bacterial culture suspension (10^8^ CFU/ml) was transferred into a well of a microtiter plate which contained 200 μl NB of different NaCl concentrations. The plate was airtight packed and incubated at 37°C for 72 h. The medium from each well of the plate was thrown out, and the well was washed 2–3 times using distilled water. A 0.01% solution of crystal violet dye was added to each dried well. After 10 min of incubation, the dye was drained, and the plate was rinsed 2–3 times with sterile distilled water (D/W). Following that, in a rinsed and dried plate, 100 μL of acetic acid (30%) was added to solubilize the cell's bound remaining dye. Absorbance at 570 nm was taken to quantify the biofilm formation.

Swarming motility was detected by adopting the method of Connelly et al. (2004). Freshly grown bacterial cultures was point-inoculated on swarm plates consisted of 0.5% Bacto-agar (w/v) and 8 g l^−1^ of NB supplemented with 5 g l^−1^ of dextrose. After 24 h of incubation at 37°C, positive results as a swarming zone was recorded.

### Characterization of different plant growth-promoting traits

The indole acetic acid (IAA) production ability of the test strain ASH15 was determined by following the standard protocol of Brick et al. (1991) using the Salkowski reagent. The phosphate solubilizing ability of the strain was analyzed by spot inoculation of the bacterial culture on the National Botanical Research Institute's Phosphate Medium agar plate (NBRIP) (Mehta and Nautiyal, 2001). The in-vitro zinc solubilization ability of the strain was carried out by employing plate assay of Sharma et al. (2012). The siderophore production ability of the test strain was assayed on a chrome azurol S (CAS) agar plate by adopting the standard method of Schwyn and Neilands (1987). The qualitative nitrogen (N) fixation capacity of the strain ASH15 was tested on an Ashby's Mannitol agar medium (Ashby, 1907). Exopolysaccharide (EPS) production was determined according to the method of Kumari et al. (2015). All the abovementioned plant growth-promoting traits were assayed under normal and different NaCl concentrations.

### Effect of strain ASH15 on sugarcane growth under greenhouse condition

To evaluate the plant growth-promoting activity of the strain ASH15, a greenhouse experiment with sugarcane under salt stress (NaCl) and non-stressed conditions was performed at the Sugarcane Research Institute, Guangxi Academy of Agricultural Sciences, Nanning, China. The bacterial inoculum was prepared by centrifugation of freshly grown bacterial cultures. The collected bacterial cell pellet was washed 2–3 times with 0.1 M phosphate buffer and resuspended in the same buffer to make the bacterial suspension. Sugarcane seedlings GT42 (15 days old), were washed 3–4 times with sterilized distilled water (D/W) and treated with bacterial suspension (CFU 10^8^) for 3 h. For control treatment, D/W was used in place of bacterial suspension. All the treated and non-treated sugarcane plants were planted in plastic pots that contained sterilized soil: sand mixture (3:1). A salt stress treatment of 200 mM NaCl was given after 10 days of plant establishment in the pot. The following treatments (T) were applied in the greenhouse experiment: T-1 (control: un-inoculated, no stress), T-2 (bacterial treatment), T-3 (200 mM salt stress treatment), and T-4 (200 mM salt stress + bacterial treatment). The experiment was conducted in a completely randomized manner in triplicate under a 16/8 h light/dark cycle with 80% field water capacity (FWC) moisture at 28 ± 2°C temperature. After 30 days of stress treatment, the plants from all treatments were uprooted, cleaned, and vegetative growth parameters, such as shoot length (SL), root length (RL), shoot fresh weight (SFW), and shoot dry weight (SDW), root fresh weight (RFW), and root dry weight (RDW), were taken.

### DNA extraction, library construction and whole genome sequencing analysis

For complete genome sequencing, genomic DNA was extracted from a full-grown culture of strain ASH15 using the DNA extraction kit (CWBIO, Beijing, China) according to the manufacturer's instructions. DNA quality and quantity were assessed using the TBS-380 fluorometer (Turner BioSystems Inc., Sunnyvale, CA, United States), and high-quality genomic DNA (OD260/280 = 1.8–2.0, >20 μg) was used for further processing. The genome sequencing was performed using single-molecule real-time (SMRT) Oxford-Nanopore and Pacbio (third generation) sequencing technology. Moreover, 15 μg of high-quality DNA was processed for fragmentation using Covaris G-TUBE (Covaris, MA, United States) for 60 s at 6,000 rpm. Genomic DNA fragments were purified, end-repaired, and ligated via SMRTbell sequencing adapters according to the manufacturer's protocol (Pacific Biosciences, CA, United States) and purified using AMPureXP beads (Beckman Coulter Genomics, MA, United States). Further, approximately 10 kb insert library was sequenced on one SMRT cell by standard procedures. For Nanopore sequencing, high-quality genomic DNA with a large fraction was selected using Blue Pippin (Sage Science, USA), followed by end-repair/dA tailing. End-repaired DNA fragments are processed for adapter ligation using a ligation sequencing kit (NBD103 and NBD114, Oxford Nanopore Technologies USA). Finally, the DNA library was quantified through Qubit 3.0 (Thermo Fisher Technologies USA). Afterward, 11 μL of DNA library was loaded into a 1 flow cell and sequenced on a PromethION sequencer (Oxford Nanopore Technologies USA).

### Genome assembly, gene prediction, and functional annotation

The raw sequence data was processed for quality checks by employing Majorbio Cloud Platform^1^ (Shanghai Majorbio Co., Ltd.). Quality-passed raw sequence data reads were then assembled into contigs using the hierarchical genome assembly method (HGAP) (Chin et al., 2013). The final genome assembly was finished using Pilon. The assembled genome was further processed for gene prediction and annotations. Prediction of coding sequence (CDS) was conducted with Glimmer version 3.02, followed by annotation using multiple databases, i.e., Pfam, Swiss-Prot, NR, Clusters of Orthologous Groups (COG), Kyoto Encyclopedia of Genes and Genomes (KEGG, http://www.genome.jp/kegg/), and Gene Ontology (GO) (Delcher et al., 2007) with Basic Local Alignment Search Tool (BLAST), DIAMOND sequence alignment and HMMER, and other tools. tRNAs and rRNAs were predicted using tRNA-scan-SE (v1.2.1) (Borodovsky and Mcininch, 1993) and Barrnap. A circular genome map of strain ASH15 was constructed using genome annotation files on the CGviewer server (Grant and Stothard, 2008).

### Taxonomic identification and phylogenetic analysis

The PCR 16S rRNA gene amplification was carried out using the universal primer pairs pA_F and pH_R (Sharma et al., 2022). The purified PCR product was sequenced using Sanger dideoxy-chain termination chemistry. The obtained sequence was assembled to make a consensus sequence by converting one strand to a reverse complement. For strain ASH15 identification, the assembled consensus 16S rRNA gene sequence was used for a BLAST (BLASTn) search against the available bacterial 16S rRNA gene sequences in the NCBI GenBank database. Further, a neighbor-joining (NJ)-based phylogenetic tree of 16S rRNA gene sequence of the strain ASH15 with 11 reference 16S rRNA gene sequences was constructed through MEGA-X. The bootstrap analysis was conducted using 1,000 replications by the Felsenstein method (Felsenstein, 1985). The evolutionary distances were calculated by the Jukes–Cantor coefficient procedure (Tamura et al., 2004).

### Identification of biosynthetic gene clusters and metabolic system analysis

The genome sequence of strain ASH15 was analyzed using antiSMASH (Blin et al., 2019) software for predicting biosynthetic gene clusters (BCGs), such as non-ribosomal peptide synthetases (NRPSs), polyketide synthases (PKSs), post-translationally modified peptides (RiPPs), hybrid lipopeptides (NRPS-PKS), and bacteriocins. Less than 70% of the amino acid identity shared by the biosynthetic Gene Clusters compared to the known clusters was considered novel. The Carbohydrate Active Enzyme Database (CAZy, http://www.cazy.org/) is a professional database for enzymes synthesizing or decomposing complex carbohydrates and sugar complexes. Carbohydrate activity enzymes derived from different species are divided into glycoside hydrolases (GHs), polysaccharide lyases (PLs), glycosyltransferases (GTs), carbohydrate esterases (CEs), auxiliary activities (AAs), carbohydrate-binding modules (CBMs), and other six major protein families.

### Additional genome analysis (sRNA prediction, repeat sequence prediction, tandem repeat prediction, and scattered repetitive sequence prediction)

Bacterial sRNA is a type of non-coding RNA with a length of 50 to 500 nt. They are located in the intergenic region of genomes, and some are in the 5′and 3′UTR regions of coding genes. We used Infernal software (http://eddylab.org/infernal/) and the Rfam database (https://rfam.xfam.org/) to predict and annotate the sRNA from the genome of strain ASH15. Tandem Repeats Finder software (Benson, 1999) was used to predict the tandem repeat sequences. Interspersed repeat, also known as a transposon element, includes DNA transposons and retrotransposons transposed by DNA-DNA. RepeatMasker software (Tarailo-Graovac and Chen, 2009) was used to identify these sequences as similar to known repetitive sequences and classify them. IslandViewer (Bertelli et al., 2017) was used to identify genomic islands in strain ASH15.

### Statistical analysis

The experimental data of the present study were subjected to analysis of variance (ANOVA) followed by DMRT (Duncan's multiple range test) with a significance level of *p* of ≤0.05 (Duncan, 1955). Bioinformatics analysis of the strain ASH15 genome was carried out through Majorbio I-Sanger (www.i-sanger.com).

## Results and discussion

### Isolation and characterization of halophilic bacteria

**Figure 1:**
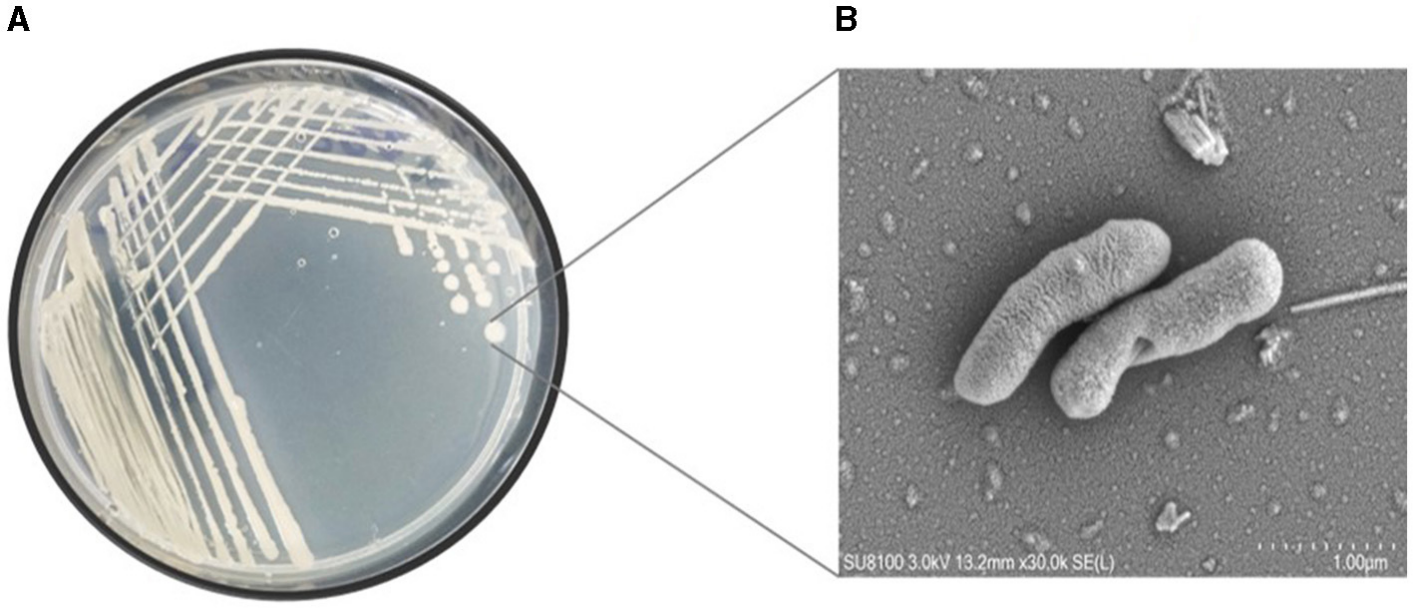
**(A)** Pure culture and **(B)** SEM micrograph of the halophilic strain *Virgibacillus halodenitrificans* ASH15 cell.

This study undertook the isolation, characterization and systematic genome analysis of a halophilic bacterial strain ASH15 and identification of their key genes that contribute in osmoadaptation (stress tolerance), plant growth-promoting (PGP) traits and other industrially important biomolecules production. A Gram-positive, spore-forming, rod-shaped, and motile halophilic bacteria strain ASH15 was isolated from the collected soil sample (Figure 1). Strain ASH15 showed medium-sized, round colonies with a ceramic-white, opaque appearance and smooth margins. Further, the carbon source utilization on GNIII MicroPlate™ (Biolog Inc., Hayward, CA), was used to extricate the metabolic sensitivity of the isolated halophilic bacterial strain ASH15 (Zhao et al., 2019). Results showed that strain ASH15 was able to metabolize a wide range of carbon sources. Strain ASH15 was positive for 24 sugars and chemically sensitive to 16 substrates, 2 hexose-PO4, 8 amino acids, 8 hexose acids, and 13 carboxylic acids, esters, and fatty acids (Supplementary Table 1). The utilization of different carbon sources by the bacterial cell assists as components of the metabolic network, whereby they are broken down to facilitate the source of amino acids and other building blocks to make up a cell (Wang et al., 2019). These metabolic properties of halophilic bacteria might lead to their response and adaptation in specific extreme environments (Sharma et al., 2021).

**Figure 2:**
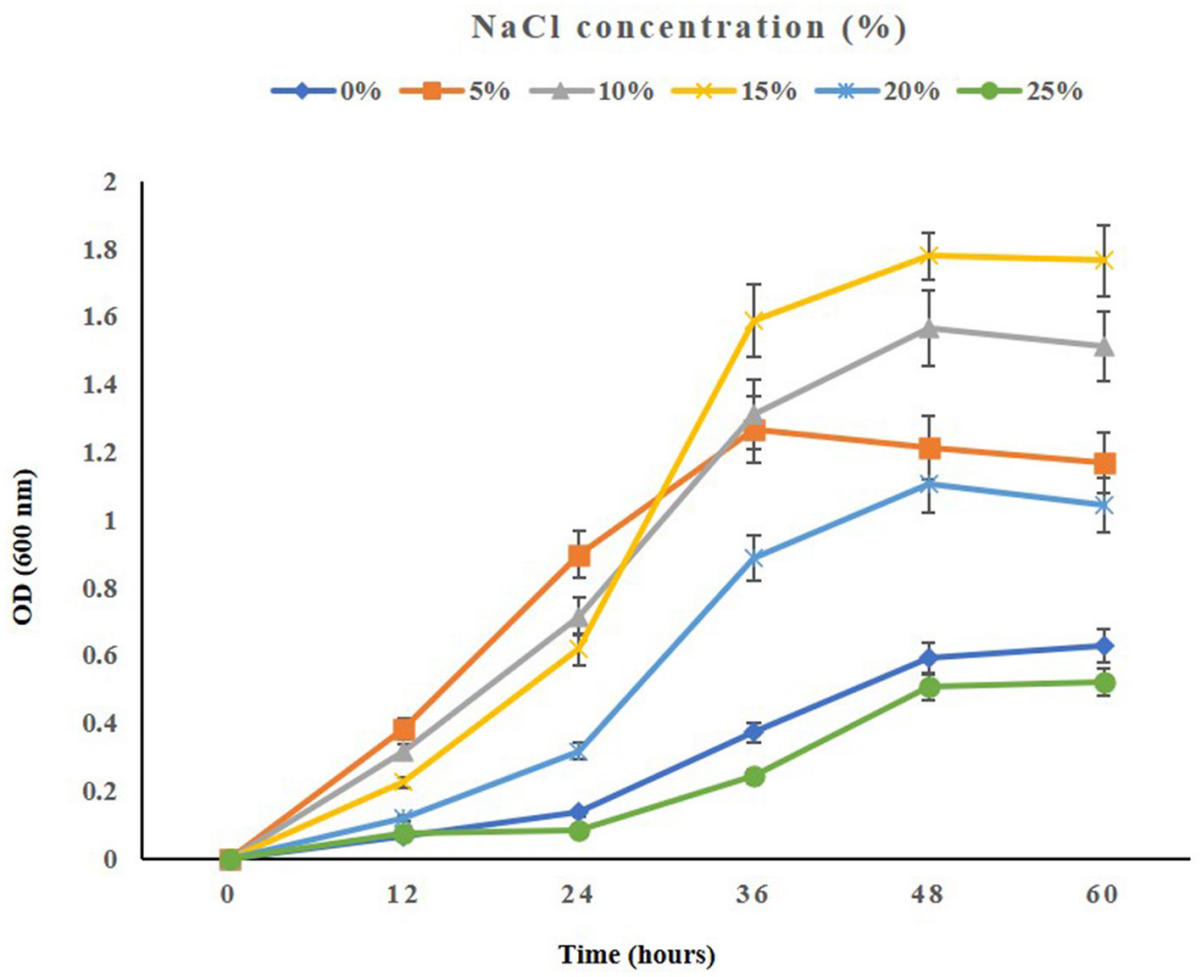
The growth pattern of the halophilic strain *Virgibacillus halodenitrificans* ASH15 at different NaCl concentrations.

When testing the growth of the strain ASH15 under various NaCl concentrations, we observed that supplementing nutrient broth with 0.5–25% NaCl had a positive effect on bacterial growth. The maximum growth (OD at 600 nm) of the strain ASH15 was observed at 15% as compared to NB with 0.5% NaCl. Further results showed that the growth of ASH15 was in contrast, slightly delayed when NaCl concentration was increased up to 25% (Figure 2). These findings demonstrate that strain ASH15 is moderately halophilic and is able to withstand up to 25% NaCl concentrations. In accordance with our findings recently, Srivastava et al. (2022) reported that *Chromohalobacter salexigens* ANJ207 was able to grow up to 30% NaCl concentration. The capacity to withstand moderate salt stress might help ASH15 survive as a free-living bacterium in saline soils.

The qualitative evaluation of plant growth-promoting attributes revealed that strain ASH15 was able to fix nitrogen, solubilize phosphate and zinc, and produce siderophore and ammonia. In addition, quantitative estimation of IAA revealed that strain ASH15 produced 54.7, 76.1, 82.8, and 80.3 μg/ml IAA at 0, 5, 10, and 15% NaCl concentrations respectively. Interestingly, strain ASH15 has all the PGP activity up to higher NaCl concentrations. Furthermore, strain ASH15 produces EPS at all the tested NaCl concentrations (Table 1). Results of the biofilm formation assay showed that strain ASH15 was able to produced biofilm in all the tested NaCl concentrations with maximum at 15% NaCl. These results showed the possible strategies that the strain ASH15 might employ on its host plant for salt stress alleviation and plant growth promotion. These findings are consistent with previous research studies, where halophilic bacteria, such as *Bacillus halophilus, Marinococcus halophilus, Halobacillus litoralis, Saliiococcus hispanicus*, and *Halobacillus halophilus*, were recognized to grow optimally between 10 and 15% NaCl concentrations (Sarwar et al., 2015; Reang et al., 2022; Srivastava et al., 2022).

**Table 1:** Plant growth promoting attributes of the strain *Virgibacillus halodenitrificans* ASH15.

**NaCl (%)**	**IAA (μg/ml)**	**P-solubilization**.	**Zn-solubilization**.	**Siderophore**	**N fixation**	**EPS (g/ml)**
0%	54.76 ± 1.8	++	+	++	+	1.66 ± 0.11
5%	76.19 ± 2.4	+++	++	+++	++	2.40 ± 0.16
10%	82.86 ± 1.5	+++	+++	++	++	2.88 ± 0.15
15%	80.43 ± 2.0	+++	+	+	++	3.29 ± 0.17

(+) production in normal level (++) production in medium level, (+++) production in high level, (-) negative for test. Values are expressed as Mean ± Standard Error.

### Phylogeny of strain ASH15

**Figure 3:**
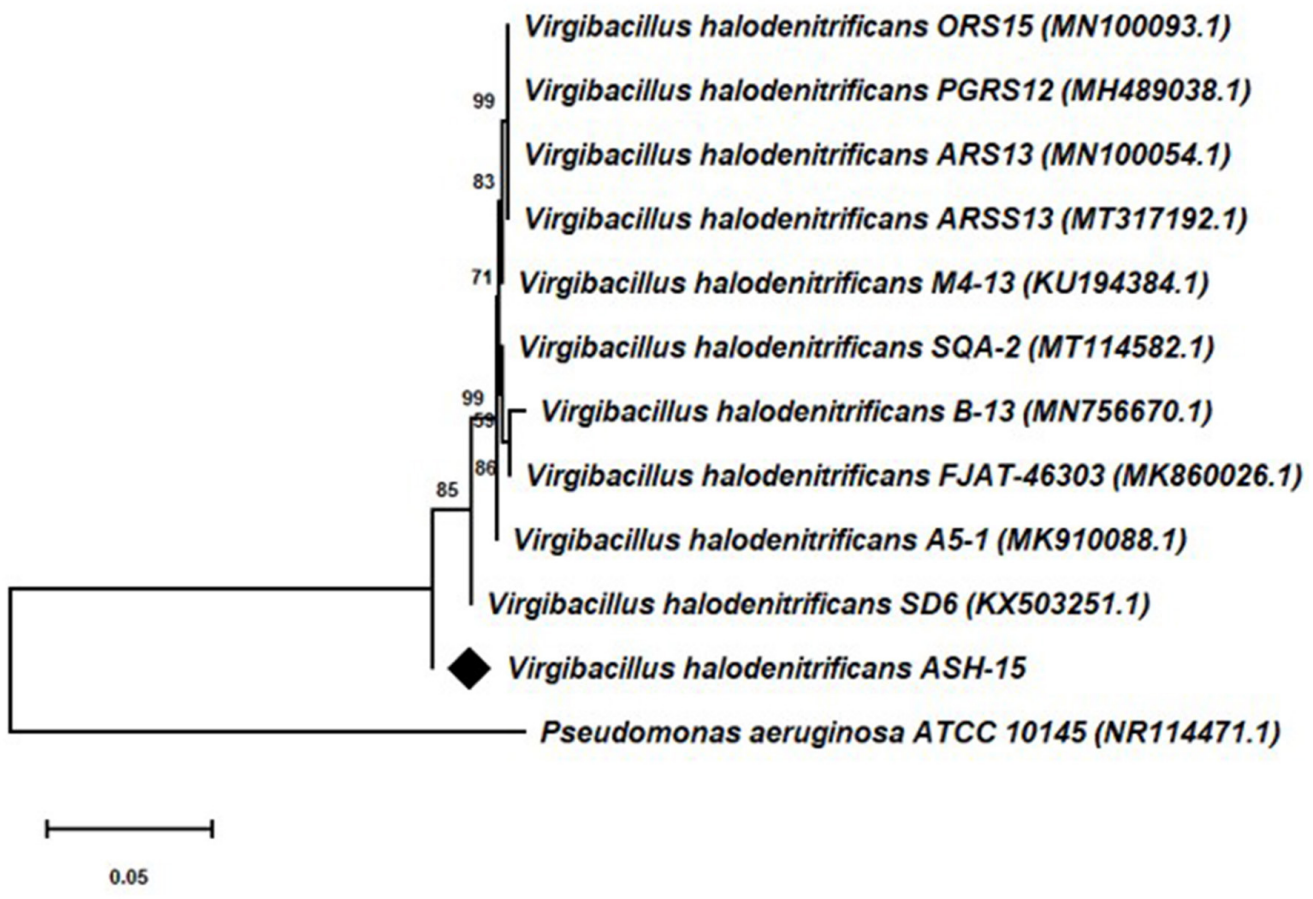
16S rRNA gene sequencing-based phylogenetic tree of the *Virgibacillus halodenitrificans* ASH15 with its nearest member of the NCBI GenBank database.

Based on the 16S rRNA gene sequencing analysis and BLASTn search, strain ASH15 showed 100% similarity with *Virgibacillus halodenitrificans* of the NCBI database. A neighbor-joining (NJ) phylogenetic tree was constructed with the similar bacterial sequences of the NCBI GenBank database (Figure 3). The phylogenetic analysis provides an important depiction of the evolutionary relationship between different strains (Srivastava et al., 2022).

### Effect of strain ASH15 on sugarcane growth under greenhouse conditions

**Figure 4:**
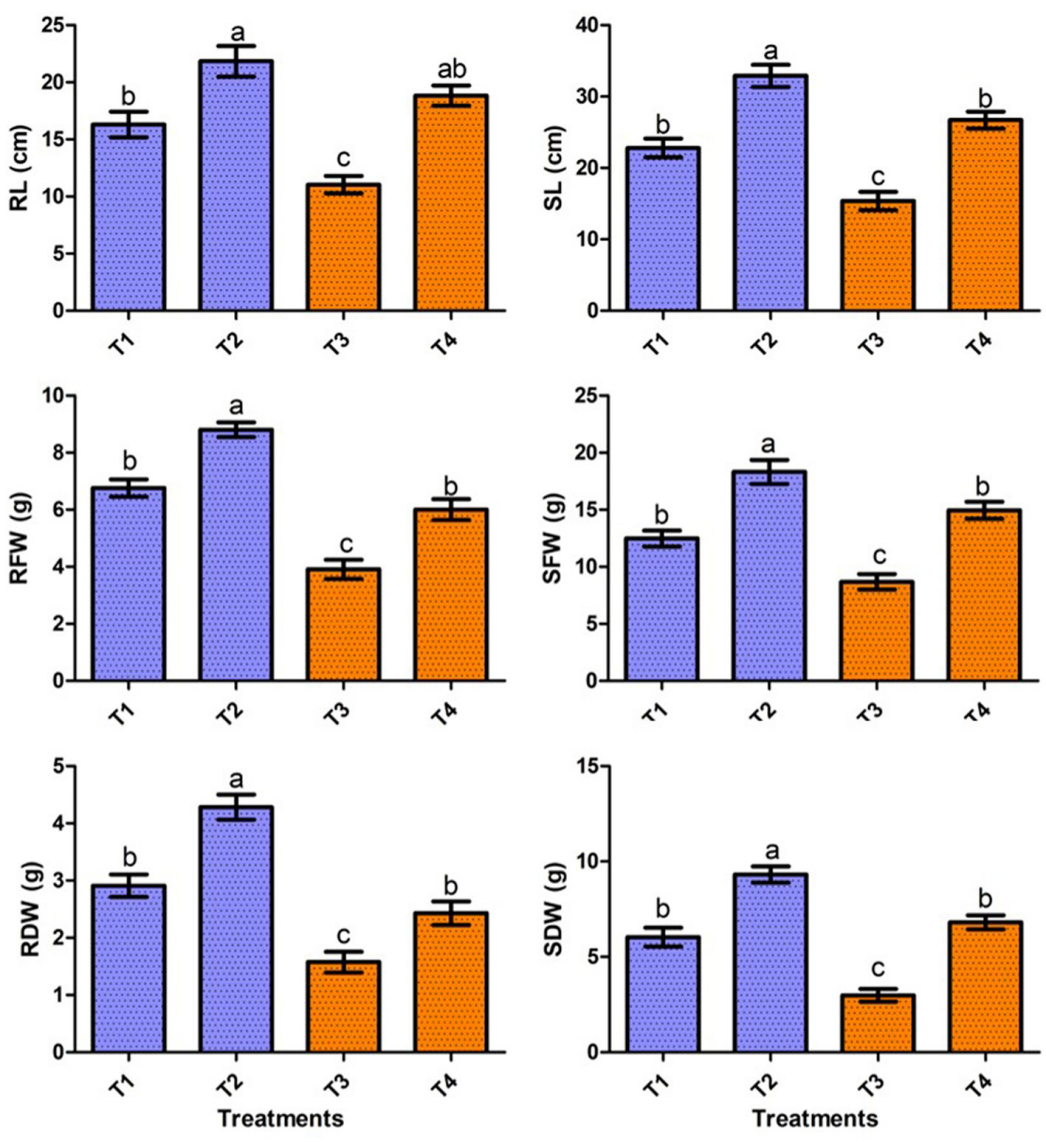
Effect of strain ASH15 on sugarcane growth under normal and NaCl (200 mM) stressed conditions. (RL, root length; RFW, root fresh weight; RDW, root dry weight; SL, shoot length; SFW, shoot fresh weight; and SDW, shoot dry weight). The figures represent the mean values ± standard error of three independent experiments. Bars indicate ± SE (standard error of mean). The letters on the bars indicate DMRT at a *p*-value of <0.05.

In the present study, the positive effect of *V. halodenitrificans* strain ASH15 on sugarcane growth under salt stress conditions was assessed under normal and salt stress (200 mM NaCl) conditions (Figure 4). Results of the greenhouse study showed that salinity stress imposes adverse effects on sugarcane vegetative growth. However, strain ASH15 application significantly (*p* < 0.05) enhanced the growth of sugarcane plants under normal as well as NaCl stress conditions (Figure 4). The results of greenhouse pot experiments showed that salt stress treatment (T-3, 200 mM NaCl) decreased root length (RL) and shoot length (SL) growth by 32.3% and 25.8%, respectively, as compared to uninoculated non-stressed (T1). Whereas, application of strain ASH15 (T-4) treatment remarkably (*p* < 0.05) enhanced the root length and shoot length by 71.2% and 64.4%, respectively, over un-inoculated NaCl-stressed plants (Figure 4). Similarly, salt stress (T-3) treatment decreases root fresh weight (RFW) and shoot fresh weight (SFW) by 42.1 % and 30.9%, respectively, over uninoculated non-stress plants (T-1). In contrast, strain ASH15 (T-4) boost up the RFW and SFW by 53.5% and 72.0%, respectively, as compared to uninoculated salt-stressed plants (T-3). Moreover, similar trends were observed in the case of root dry weight (RDW) and shoot dry weight (SDW), where salt stress (T-3) reduced the RDW and SDW by 45.8% and 57.6%, respectively, over their uninoculated control (T-1). However, strain ASH15 (T-4) increased the RDW and SDW by 54.1% and 109.1%, respectively, compared to the uninoculated salt-stressed control (T-3) (Figure 4). The results of the pot experiment demonstrated that the sugarcane plant's overall health, growth, and development were reliant on the presence of strain ASH15, which regulated an adequate amount of multiple plant nutrient levels (Alishahi et al., 2020; Khumairah et al., 2022). Therefore, in this study, we explored the strain ASH15 genome and mined the gene codes for almost all PGP traits like IAA, nitrogen fixation, phosphate solubilization, and siderophore production. Sultana et al. (2020) recently reported that salt-tolerant bacteria significantly increased rice plant growth. The results are also in accordance with those reported by Bhattacharyya et al. (2017), Asaf et al. (2018), and Abdullahi et al. (2021), where they analyzed the presence of multiple genes encoding for PGP mechanisms in plant growth-promoting bacteria.

### Genome analysis of *V. halodenitrificans* ASH15

**Figure 5:**
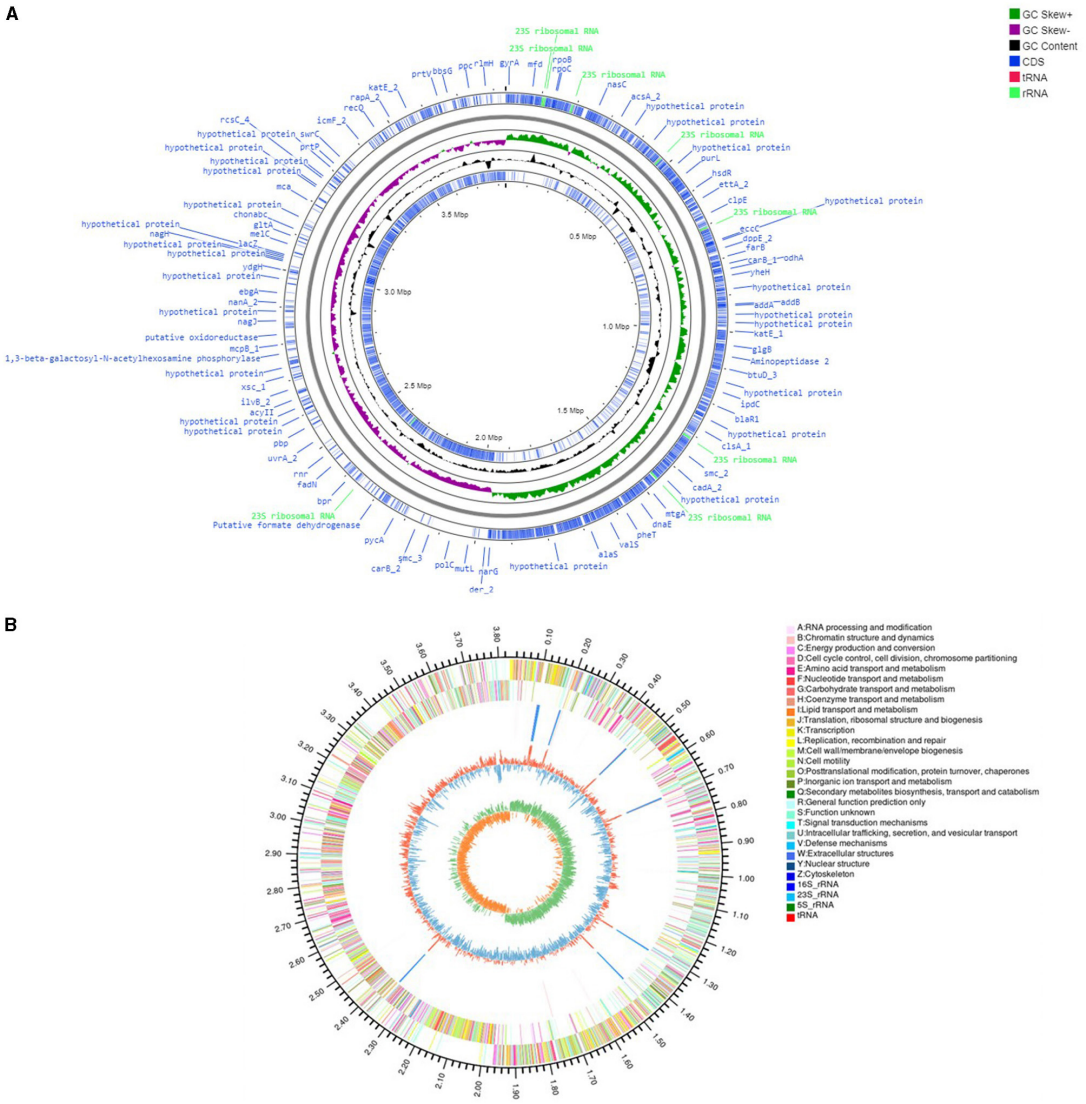
**(A)** Circular representation of the chromosome/genome of *Virgibacillus halodenitrificans* strain ASH15. The Outermost and innermost rings represent two strands of the genome. The outermost ring displays the functional annotation of genes (CDS), tRNA, and rRNA distribution in the genome. The third ring (black in color) shows the GC content of the genome, while the fourth ring (green in color) represents the GC skewness throughout the genome. **(B)** A–Z, the outer two circles, show the functional classification of the CDS genes in the chromosome with the colors of the COG. The inner circle shows rRNAs and tRNAs.

**Figure 6:**
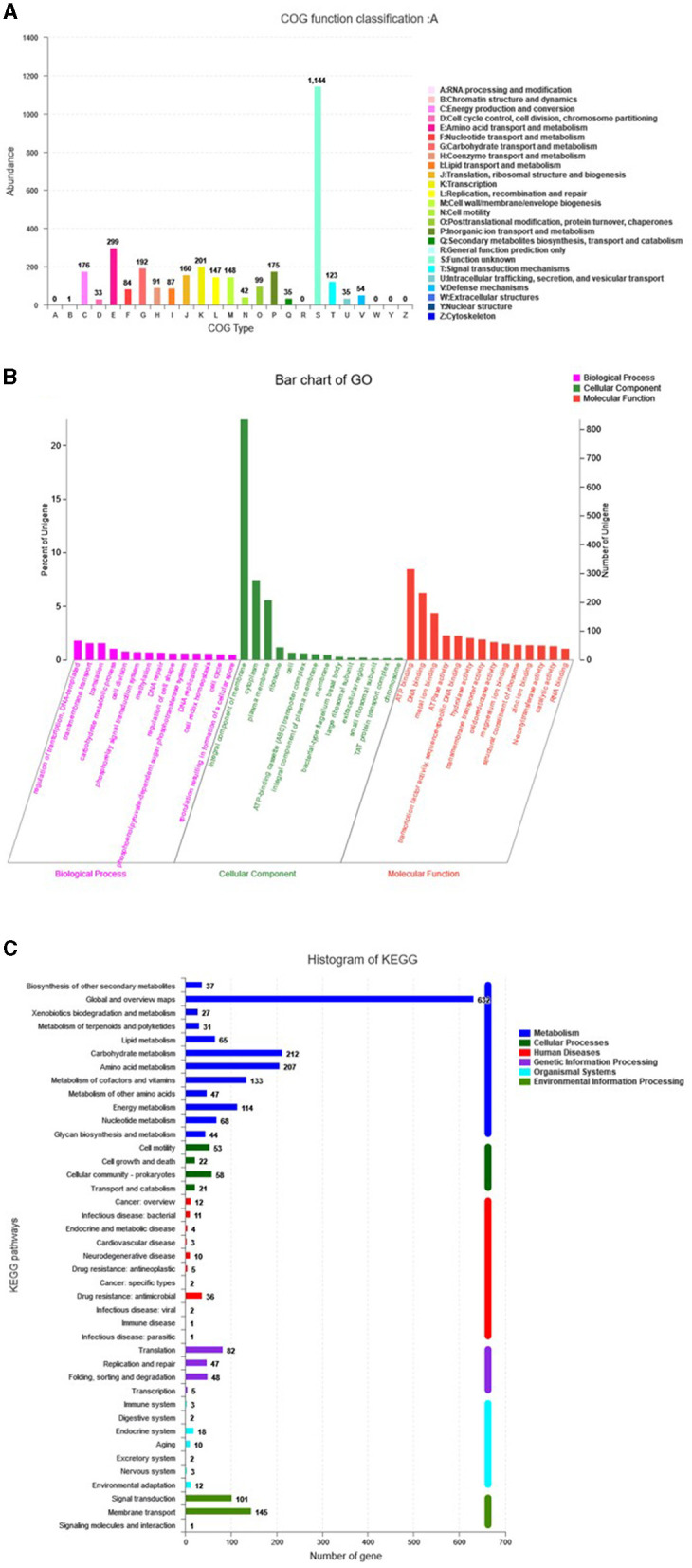
Predicted genes of the *Virgibacillus halodenitrificans* strain ASH15 genome through **(A)** COG, **(B)** GO, and **(C)** KEGG databases.

The genome assembly details of the strain ASH15 are given in Table 2. The high-quality raw sequence data was assembled with a hybrid genome assembly, and a single scaffold was achieved. The genome of *V. halodenitrificans* strain ASH15 is composed of a circular chromosome (Figure 5) of 3,832,903 base pairs with an average G+C content of 37.54%. There was no plasmid identified in the genome assembly. The genome was processed for gene prediction, and the total predicted genes were 3,807, which included ~3,721 protein-coding genes (CDS), 62 tRNAs, and 24 rRNA genes (Table 2). CDS constitute 3,207,696 bases (83.69%) of the genome, with an average gene length of 862.05 bases. Approximately 10.78% (803483 bases) of the genome was found to be intergenic. Furthermore, predicted genes against various databases were characterized. The number of COG genes, Gene Ontology (GO), KEGG, NR (Non-redundant Protein Database), and SwissProt were 3,268, 2,575, 2,008, 3,644, and 2,683 respectively (Table 2 and Figures 6A–C). The complete genome sequence of the strain *V. halodenitrificans* ASH15 has been deposited at the NCBI/GeneBank with accession number CP090006.

**Table 2:** Genome characteristics of *Virgibacillus halodenitrificans* strain ASH15.

**Characteristics**	**Value**
Genome size (bp)	3,832,903
Chromosome	1
Chromosome size (bp)	3,832,903
GC content (%)	37.54
Topology	Circular
tRNA	62
rRNAs (5S, 16S, 23S)	24
CDS	3,721
CDS (bp)	3,207,696
CDS (% of genome)	83.69
Average gene length (bp)	862.05
Intergenic region (bp)	803,483
Intergenic region (%)	10.78
Genomic islands	7
CRISPR	36
Insertion sequences	9
Genes annotated with COG	3,326
Genes annotated with GO	2,575
Genes annotated with KEGG	2,008
Genes annotated with NR	3,644
Genes annotated with Swiss-Prot	2,683

### Genetic potential of various stress tolerance in the ASH15 genome

Halophilic bacteria have unique inherent osmoadaptation mechanisms for stress adaptation, which could be used for agriculture, food, and fermentation industries (Gunde-Cimerman et al., 2018; Vaidya et al., 2018). Genome analysis of the strain ASH15 confirmed the presence of several key genes responsible for different abiotic stress tolerances, mainly osmotic stress (*proVWXSBA, fadANM, betBA, trkAH, opuBDCA, opcR, putP, yrgG*, kch, and *nhaC*), ectoine biosynthesis (*ectCBAD*), and oxidative stress *(hmp, pfpI*, Usp*, katE*, and *osmC*) (Table 3). These osmolytes, or compatible solutes, provide osmotic balance to the bacteria without disturbing their cell functions (Roberts, 2005; León et al., 2018).

**Table 3:** Genes associated with abiotic stress responses in *Virgibacillus halodenitrificans* strain ASH15.

**Stress**	**Gene IDs**	**Gene annotations**	**GO IDs**	**Chromosome location**
**Osmotic stress**				
Proline	*proV*	Glycine betaine/proline transport system ATP-binding protein	GO:0031460	891274-892473
	*proW*	Glycine betaine/proline transport system permease protein	GO:0055085	892463-893317
	*proX*	Glycine betaine/proline transport system substrate-binding protein	GO:0043190	893428-894330
	*proX*	Glycine betaine/proline transport system substrate-binding protein	GO:0043190	894427-895362
	*proB*	Glutamate 5-kinase	GO:0055129	768476-769576
	*proA*	Glutamate-5-semialdehyde dehydrogenase	-	769590-770837
	*proX*	Glycine betaine/proline transport system substrate-binding protein	-	3749454-3748555
	*betB*	Betaine-aldehyde dehydrogenase	GO:0019285	3749899-3751371
	*betA*	Choline dehydrogenase	GO:0019285	3751639-3753324
	*proS*	Prolyl-tRNA synthetase	GO:0006433	403135-404574
	*putP*	Sodium/proline symporter	GO:0015824	573152-574672
	*putP*	Sodium/proline symporter	GO:0015824	2279082-2280548
	*yrbG*	Sodium/calcium exchanger protein;	GO:0055085	284640-285590
	*panF*	Sodium/panthothenate symporter	GO:0036376	318578-320014
	*cvrA*	K(+)/H(+) antiporter NhaP2	GO:0006813	3592714-3594207
	*kch*	Potassium channel family protein	GO:0008076	195811-195101
	*lctB*	Two pore domain potassium channel family protein	GO:0016021	695254-695685
	*trkA*	trk system potassium uptake protein C	GO:0006813	2255550-2254885
	*trkH*	trk system potassium uptake protein D	GO:0016021	2282378-2281014
	*nhaC*	Na(+)/H(+) antiporter NhaC	GO:0016021	2821802-2820342
	*fadA*	Acetyl-CoA acyltransferase	GO:0016747	2442609-2441434
	*fadN*	3-hydroxyacyl-CoA dehydrogenase	-	2445023-2442624
	*fadM*	Proline dehydrogenase	GO:0010133	2446131-2445217
Ectoine	*ectC*	L-ectoine synthase	GO:0019491	2330170-2329784
	*ectB*	Diaminobutyrate-2-oxoglutarate transaminase	GO:0019491	2331484-2330207
	*ectA*	L-2,4-diaminobutyric acid acetyltransferase	GO:0019491	2332015-2331503
	*ectD*	Ectoine dioxygenase	GO:0016706	3474559-3473663
Glycine/betaine	*opuD*	Glycine betaine transporter	GO:0071705	323757-325247
	*opuD*	Glycine betaine transporter	GO:0071705	505199-506722
	*opuC*	Osmoprotectant transport system substrate-binding protein	GO:0043190	1990166-1988640
	*opuA*	Osmoprotectant transport system ATP-binding protein	GO:0005524	1991224-1990163
	*opuBD*	Osmoprotectant transport system permease protein	GO:0055085	2334871-2334209
	*opuC*	Osmoprotectant transport system substrate-binding protein	GO:0043190	2335802-2334873
	*opuBD*	Osmoprotectant transport system permease protein	GO:0055085	2336463-2335819
	*opuA*	Osmoprotectant transport system ATP-binding protein	GO:0031460	2337636-2336476
	*opcR*	HTH-type transcriptional regulator, osmoprotectant uptake regulator	GO:0003677	2337857-2338429
	*opuD*	Glycine betaine transporter	GO:0071705	2727709-2726204
Oxidative stress	*hmp*	Nitric oxide dioxygenase	GO:0009636	37386-36160
	*pfpI*	General stress protein 18	GO:0008233	38034-38549
	*Usp*	Universal stress protein	-	309979-309560
	*katE*	Catalase	GO:0006979	470556-469066
	*osmC*	Response to oxidative stress	GO:0006979	2158630-2158211
**Cold/heat stress**				
	*cspA*	Cold shock-like protein CspC	GO:0005737	417715-417912
	*cspA*	Cold shock-like protein CspLA	GO:0005737	616606-616806
	*cspA*	Cold shock-like protein CspD	GO:0005737	1943998-1943798
	*hrcA*	Heat-inducible transcription repressor HrcA	GO:0045892	1716523-1717557
	*-*	Participates actively in the response to hyperosmotic and heat shock	GO:0006457	1717638-1718198
	*dnaK*	Heat shock protein	GO:0006457	1718231-1720072
	*-*	Heat induced stress protein YflT	-	2302479-2302829
	*HSP20;*	Small heat shock protein C4	-	2356669-2357109
	*htpX*	Protease HtpX	GO:0016021	3565876-3566754
	*htpG*	Chaperone protein HtpG	GO:0006457	80666-78786
	*ctsR*	Transcriptional regulator CtsR	GO:0006355	113128-113595
	*-*	Hsp20/alpha crystallin family	-	543367-542930
**Antibiotic stress**				
	*-*	Tetracycline resistance protein	-	126052-126561
	*norM*	Probable multidrug resistance protein NorM	-	838660-837296
	*bacA*	Bacitracin resistance protein BacA	GO:0008360	957794-956973
	*lmrB*	Lincomycin resistance protein LmrB	GO:0016021	1117314-1115830
	*fsr*	Fosmidomycin resistance protein	GO:0016021	2811379-2810162
	*ykkD*	Probable guanidinium efflux system subunit GdnD	GO:0016021	3328577-3328263
	*ykkC*	Probable guanidinium efflux system subunit GdnC	GO:0016021	3328918-3328577
	*yitG*	MFS transporter, ACDE family, multidrug resistance protein	-	3597908-3599146
	*pbp1b*	Penicillin-binding protein	-	1491396-1488400
**Heavy metals**				
	*czcD*	Cobalt-zinc-cadmium efflux system protein	GO:0016021	2264058-2264960
	*ecfA1*	ABC transporter	GO:0055085	161202-162041
	*ecfA2*	ABC transporter;AAA domain, putative AbiEii toxin, Type IV TA system	-	162017-162889
	*ecfT*	Cobalt transport protein	GO:0016021	162882-163682
	*zupT*	Zinc transporter, ZIP family	GO:0016021	1393351-1394172
	*corA*	Magnesium transporter	GO:0016021	1387297-1386344
	*yqgT*	Zinc carboxypeptidase	GO:0008270	1955473-1954283
	*rseP*	Zinc metalloprotease RasP	GO:0016021	2071722-2070457
	*czrA*	Zinc-responsive transcriptional repressor	GO:0003700	2263738-2264040
	*czcD*	Cobalt-zinc-cadmium efflux system protein	GO:0016021	2264058-2264960
	*yogA*	Zinc-binding alcohol dehydrogenase/oxidoreductase	-	2292089-2291109
	*ftsH*	ATP-dependent zinc metalloprotease FtsH	GO:0051301	90304-92340
	*arsR*	Transcriptional regulator	GO:0003700	1378392-1378730
	*arsB*	Arsenic transporter	GO:0046685	1378746-1380044
	*arsC*	Arsenate reductase (glutaredoxin)	GO:0046685	1380063-1380482
	*znuA*	Zinc transport system substrate-binding protein	GO:0030001	2449475-2450401
	*znuC*	Zinc transport system ATP-binding protein	GO:0005524	2450418-2452053
	*znuB*	Zinc transport system permease protein	GO:0043190	2451190-2452456
	*zurR*	Fur family transcriptional regulator, zinc uptake regulator	GO:0003677	2452043-2452827
	*nprE*	Zinc metalloprotease	GO:0005576	3186475-3184820
	*qor*	Zinc-binding dehydrogenase	GO:0016491	3671355-3670381
	*sprL*	Zinc ion binding	GO:0005737	470890-471348
	*zntA*	Zn2+/Cd2+-exporting ATPase	GO:0016021	661474-659534
Manganese	*mntC*	Manganese transport system substrate-binding protein	GO:0030001	24740200-2473076
	*mntB*	Manganese transport system permease protein	GO:0043190	2474910-2474053
	*mntA*	Manganese transport system ATP-binding protein	GO:0005524	2475650-2474907
Copper	*copZ*	Copper chaperone	GO:0030001	2446469-2446263
	*copA*	P-type Cu+ transporter	GO:0030001	2448887-2446497
	*csoR*	CsoR family transcriptional regulator, copper-sensing transcriptional repressor	GO:0006355	2449203-2448901
	*copB*	Copper-exporting P-type ATPase B	GO:0016021	1405364-1407472
	*cutC*	Copper homeostasis protein cutC	-	3246050-3245355
	*ycnK*	Copper-sensing transcriptional repressor	GO:0003677	3641001-3640405
Molybdenum	*modA*	Molybdate transport system substrate-binding protein	GO:0015689	3628744-3629541
	*modB*	Molybdate transport system permease protein	GO:0016021	3629557-3630246
	*modC*	Molybdate transport system ATP-binding protein	GO:0005524	3630272-3630889
**Fluoride stress**				
	*crcB*	Fluoride exporter	-	2452441-2452827
	*crcB*	Fluoride exporter	GO:0005887	2452824-2453159

In addition, further analysis revealed that the strain ASH15 genome has various other abiotic stress tolerance genes such as cold-shock protein (*cspA*), heat shock proteins (*hrcA, dnaK*, hsp20, *htpX, htpG*, and *ctsR*), heavy metals such as arsenic (*arsRBC*), cobalt (*czcD, ecfT, ecfA1, ecfA2*), zinc (*zupT, yqgT, rseP, czrA, znuACB, zurR, nprE, qor, sprL*), cadmium (*zntA*), magnesium (*corA*), molybdenum (*modABC*), copper (*copZA, csoR, copB, cutC, ycnK*), and manganese (*mntCBA*), antibiotics (*norM, bacA, lmrB, fsr, pbp1b, ykkDC*, and *yitG*), and fluoride resistance (*crcB*) (Table 3). These groups of genes provide stress-tolerant capabilities to strain ASH15 and enable it to survive in extreme conditions. Fluoride exporter genes, *crcB*, were involved in multilevel stress responses (Calero et al., 2022). Several PGPR genera have been described to succeed in heavy metal stresses to improve plant tolerance, especially abiotic stresses, and crop yields (Tiwari and Lata, 2018). These proteins are linked to tolerance to cobalt, zinc, copper, arsenic and cadmium (Kang et al., 2020).

### Genes related to plant growth promotion in the strain ASH15

Sugarcane is a long-term economically important crop, and for its growth, various types of plant nutrients such as N, P, K, and phytohormones are required. Thus, to decrease the application of chemical fertilizers in the current era, PGPR with various PGP attributes promises an alternative approach to plant nutrient requirements (Sharma et al., 2021). Therefore, in the present study, we revealed the plant growth promotion potential of *V. halodenitrificans* ASH15. The genome of the strain covers so many genes that encode various PGP traits, such as phytohormone IAA production, nitrogen fixation, phosphate solubilization, ammonia assimilation, and siderophore production (Table 4, Guo et al., 2020).

**Table 4:** Genes associated with PGP traits in *Virgibacillus halodenitrificans* strain ASH15.

**PGP traits**	**Gene IDs**	**Gene annotations**	**GO IDs**	**Chromosome location**
**Nitrogen metabolism**				
	*narK*	MFS transporter, NNP family, nitrate/nitrite transporter	GO:0016021	259339-260835
	*nreB*	Two-component system, NarL family, sensor histidine kinase NreB	GO:0016021	260896-261615
	*nreC*	Two-component system, NarL family, response regulator NreC	GO:0006355	261612-262265
	*nreA*	Nitrogen regulatory protein A	-	262255-262725
	*nos*	Nitric oxide synthase oxygenase	GO:0006809	519663-520754
**Nitrogen fixation**	*nifU*	Putative nitrogen fixation protein	GO:0016226	2409694-2409918
	*iscU*	Nitrogen fixation protein NifU and related proteins	GO:0016226	2430245-2429811
	*norG*	GntR family transcriptional regulator, regulator for abcA and norABC	GO:0009058	618000-619412
	*narG*	Nitrate reductase, alpha subunit	GO:0042126	1957499-1961182
	*narH*	Nitrate reductase, beta subunit	GO:0042126	1961172-1962665
	*narJ*	Nitrate reductase molybdenum cofactor assembly chaperone NarJ/NarW	GO:0051131	1962670-1963272
	*narI*	Nitrate reductase gamma subunit	GO:0016021	1963286-1963975
**Ammonia assimilation**				
	*gltX*	Glutamate–tRNA ligase	GO:0006424	120934-122403
	*gltA*	Citrate synthase	GO:0005737	1541961-1543076
	*gltS*	Glutamate:Na+ symporter, ESS family	-	2322419-2320896
	*glnQ*	Glutamine transport system ATP-binding protein	GO:0005524	2721456-2720734
	*glnP*	Glutamine transport system permease protein	GO:0071705	2722112-2721453
	*glnH*	Glutamine transport system substrate-binding protein	GO:0016020	2723108-2722317
	*glnR*	MerR family transcriptional regulator, glutamine synthetase repressor	GO:0006355	2011319-2010936
	*glnA*	Glutamine synthetase	GO:0006542	3089088-3087751
	*gltD*	Glutamate synthase [NADPH] small chain	GO:0006537	3090618-3089122
	*gltB*	Glutamate synthase [NADPH] large chain	GO:0006537	3095251-3090698
	*gltC*	HTH-type transcriptional regulator GltC	GO:0003677	3095376-3096278
	*gdhA*	Glutamate dehydrogenase	GO:0006520	2300858-2299479
	*asnB*	Asparagine synthetase	GO:0006541	3096385-3098229
	*pyrG*	CTP synthase	GO:0006541	3404454-3402853
**Phosphate metabolism**				
	*ppaC*	Manganese-dependent inorganic pyrophosphatase	GO:0005737	727813-728745
	*phoA*	Alkaline phosphatase	GO:0016791	627314-625959
	*phoB1*	Two-component system, OmpR family, alkaline phosphatase synthesis response regulator PhoP	GO:0006355	1546461-1547156
	*phoR*	Two-component system, OmpR family, phosphate regulon sensor histidine kinase PhoR	GO:0006355	1547153-1548529
	*phoH*	Phosphate starvation-inducible protein PhoH and related proteins	GO:0005524	1730983-1731942
	*pstS*	PstS family phosphate ABC transporter substrate-binding protein	GO:0042301	3588504-3589496
	*pstC*	Phosphate ABC transporter permease subunit PstC	GO:0006817	3589588-3590541
	*pstA*	Phosphate ABC transporter permease PstA	GO:0035435	3590544-3591428
	*pstB*	Phosphate import ATP-binding protein PstB	GO:0005886	3591505-3592341
**Potassium transport**				
	*cvrA*	Potassium/proton antiporter	GO:0006813	3592714-3594207
**Siderophore**				
	*-*	ABC transporter permease	GO:0016021	375879-376832
	*-*	Iron chelate uptake ABC transporter family permease subunit	GO:0016021	376822-377772
	*-*	ABC transporter ATP-binding protein	GO:0005524	377766-378521
	*-*	ABC transporter substrate-binding protein	GO:0006826	378831-379808
	*-*	Iron export ABC transporter permease subunit fetb	GO:0016021	507556-506792
	*perR*	Ferric uptake regulator, Fur family	GO:0003677	698241-698684
	*-*	Iron complex transport system substrate-binding protein	-	1096047-1097045
	*-*	Iron complex transport system permease protein	-	1097017-1098075
	*-*	Iron complex transport system ATP-binding protein	-	1098075-1099562
	*fhuB*	Iron ABC transporter permease	GO:0016021	2308243-2306216
	*fhuD*	Iron-siderophore ABC transporter substrate-binding protein	-	2309249-2308248
	*-*	Iron export ABC transporter permease subunit FetB	GO:0016021	2748819-2749649
	*FecCD*	Iron ABC transporter permease	GO:0016021	3012286-3011234
	*afuB*	Ferric transport system permease protein	GO:0055085	2962193-2960523
	*afuC*	Fe(3+) ions import ATP-binding protein	-	2963319-2962156
	*afuA*	Extracellular solute-binding protein	-	2964360-2963344
	*-*	ABC transporter ATP-binding protein	GO:0005524	3011187-3010357
	*-*	Iron ABC transporter permease	GO:0016021	3012286-3011234
	*-*	Iron ABC transporter permease	GO:0016021	3013281-3012283
	*-*	ABC transporter substrate-binding protein	-	3013486-3014466
	*afuB*	Iron ABC transporter permease	GO:0055085	3191977-3190292
	*afuC*	ABC transporter ATP-binding protein	GO:0043190	3193081-3191978
	*afuA*	Iron ABC transporter substrate-binding protein	-	3194230-3193124
	*-*	Iron(3+)-hydroxamate import ATP-binding protein FhuC	GO:0005524	3687309-3688151
	*-*	Iron(3+)-hydroxamate-binding protein FhuD	-	3688105-3689034
	*-*	Iron(3+)-hydroxamate import system permease protein FhuB	GO:0016021	3689118-3690314
	*-*	Iron(3+)-hydroxamate import system permease protein FhuG	GO:0016021	3690311-3691318
**Plant hormones**				
**IAA biosynthesis**	*trpA*	Tryptophan synthase alpha chain	-	338845-338054
	*trpB*	Tryptophan synthase beta chain	GO:0004834	340051-338846
	*trpF*	Phosphoribosylanthranilate isomerase	-	340649-340020
	*trpC*	Indole-3-glycerol phosphate synthase	GO:0000162	341436-340639
	*trpD*	Anthranilate phosphoribosyltransferase	GO:0000162	342462-341437
	*trpG*	Anthranilate synthase component II	GO:0006541	343045-342446
	*trpE*	Anthranilate synthase component I	GO:0000162	344426-343026
	*trpS*	Tryptophanyl-tRNA synthetase	GO:0006436	965341-964346
**Root colonization chemotaxis, motility, biofilm**	*fliA*	RNA polymerase sigma factor for flagellar operon FliA	GO:0006352	2078804-2078028
	*cheD*	Chemotaxis protein CheD	GO:0006935	2079416-2078919
	*cheC*	Chemotaxis protein CheC	GO:0016787	2080044-2079409
	*cheW*	Purine-binding chemotaxis protein CheW	GO:0007165	2080510-2080049
	*cheB*	Two-component system, chemotaxis family, protein-glutamate methylesterase/glutaminase	GO:0006935	2081589-2080549
	*flhG*	Flagellar biosynthesis protein flhG	-	2082459-2081596
	*flhF*	Flagellar biosynthesis protein flhF	GO:0044781	2083573-2082452
	*flhA*	Flagellar biosynthesis protein flhA	GO:0044780	2085603-2083570
	*flhB*	Flagellar biosynthetic protein flhB	GO:0044780	2086702-2085623
	*fliR*	Flagellar biosynthetic protein fliR	GO:0044780	2087483-2086704
	*fliQ*	Flagellar biosynthetic protein fliQ	GO:0044780	2087756-2087487
	*fliP*	Flagellar biosynthetic protein fliP	GO:0009306	2088457-2087792
	*fliOZ*	Flagellar protein fliO/fliZ	GO:0044781	2089091-2088450
	*cheY*	Two-component system, chemotaxis family, chemotaxis protein cheY	GO:0000160	2089468-2089106
	*fliNY*	Flagellar motor switch protein fliN/fliY	GO:0071973	2090624-2089488
	*fliM*	Flagellar motor switch protein fliM	GO:0071973	2091612-2090614
	*fliL*	Flagellar flil protein	GO:0071973	2092064-2091645
	*flbD*	Flagellar protein flbD	-	2092272-2092057
	*flgE*	Flagellar hook protein flgE	GO:0071973	2093179-2092319
	*-*	Flagellar protein	-	2093634-2093263
	*flgD*	Flagellar basal-body rod modification protein flgD	-	2094104-2093655
	*fliK*	Flagellar hook-length control protein fliK	-	2095376-2094114
	*fliJ*	Flagellar fliJ protein	GO:0071973	2096441-2095995
	*fliI*	Flagellum-specific ATP synthase	GO:0071973	2097760-2096447
	*fliH*	Flagellar assembly protein fliH	-	2098545-2097757
	*fliG*	Flagellar motor switch protein fliG	GO:0071973	2099524-2098511
	*fliF*	Flagellar M-ring protein fliF	GO:0071973	2101132-2099537
	*fliE*	Flagellar hook-basal body complex protein fliE	GO:0071973	2101495-2101190
	*flgC*	Flagellar basal-body rod protein flgC	GO:0071973	2101958-2101512
	*flgB*	Flagellar basal-body rod protein flgB	GO:0071973	2102351-2101962
	*motA*	Chemotaxis protein motA	GO:0016021	1483620-1484444
	*motB*	Chemotaxis protein motB	GO:0016021	1484434-1485222
	*pilB*	Type IV pilus assembly protein pilB	-	1517804-1519432
	*pilT*	Twitching motility protein pilT	GO:0005524	1519445-1520485
	*pilC*	Type IV pilus assembly protein pilC	GO:0009306	1520488-1521687
	*pilA*	Type IV pilus assembly protein pilA	-	1521849-1522235
	*pilM*	Type IV pilus assembly protein pilM	-	1523108-1524025
	*hofN*	Pilus assembly protein hofN	GO:0016021	1524038-1524589
	*-*	Pilus assembly protein, pilO	GO:0016021	1524570-1525112
	*fliT*	Flagellar protein fliT	-	2541582-2541229
	*fliS*	Flagellar protein fliS	GO:0044780	2541983-2541582
	*flaG*	Flagellar protein flaG	-	2542403-2542038
	*fliW*	Flagellar assembly factor fliW	GO:0044780	2543144-2542704
	*flgL*	Flagellar hook-associated protein 3 flgL	GO:0071973	2544659-2543790
	*flgK*	Flagellar hook-associated protein 1 flgK	GO:0071973	2546184-2544670
	*fglN*	Flagellar protein flgN	GO:0044780	2546703-2546209
	*flgM*	Negative regulator of flagellin synthesis flgM	GO:0045892	2546978-2546718
	*cheY*	Two-component system, chemotaxis family, chemotaxis protein cheY	GO:0000160	2590918-2590562
	*cheW*	Purine-binding chemotaxis protein cheW	GO:0007165	2591400-2590918
	*cheA*	Two-component system, chemotaxis family, sensor kinase cheA	GO:0006935	2593417-2591411
	*cheX*	Chemotaxis protein cheX	-	2593903-2593442
	*motB*	Chemotaxis protein motB	GO:0016020	2594695-2593919
	*motA*	Chemotaxis protein motA	GO:0016021	2595473-2594682
	*fliD*	Flagellar hook-associated protein 2	GO:0071973	2600255-2598189

Indole-3-acetic acid (IAA) is an important phytohormone involved in various physiological processes, including cell enlargement and division, tissue differentiation, and responses to light and gravity. The ability to synthesize IAA is a well-characterized trait in halophilic PGPR (Pérez-Inocencio et al., 2022). Bacterial IAA is involved in overcoming stress, serving as a C/N source, and playing a role in plant–microbe interactions (Defez et al., 2019). In the current study, we observed that strain ASH15 was capable of synthesizing IAA, and its genome consists of *trp*A, *trpB, trpC, trpD, trpE, trpF, trpG*, and *trpS* genes, which code for enzymes of the IAA biosynthesis pathway (Table 4). Similar to our findings, tryptophan biosynthesis genes (*trpABD*) are involved in IAA production in *Sphingomonas* sp. LK11 (Asaf et al., 2018).

Another strategy of PGPR to enhance plant growth is to fix atmospheric nitrogen. Nitrogen is an essential nutrient element for soil fertility, sugarcane plant growth and development, physiological and metabolic activities, and sustainable sugarcane crop production (Singh et al., 2022). PGPR catalyzes nitrogen fixation through the *nif* (nitrogenase complex) gene-coded nitrogenase enzyme. In this study, the strain ASH15 genome lacks genes (*nifDHK*) coding the nitrogenase enzyme, but contains genes related to dissimilatory nitrate reduction (Table 4). These include narGHIJ, a nitrate/nitrite ABC transporter (narK), a putative nitrogen fixation protein (nifU), and various other genes associated with nitrogen metabolism and transport (*iscU, norG, nreBCA*, and nos). Strain ASH15 also has genes coding for ammonia assimilation, such as *gltXASDBC, glnQPHRA, gdhA, asnB*, and *pyrG* (Table 4). These results showed that strain ASH15 is able to incorporate nitrate and nitrite for assimilation into ammonia and can incorporate ammonia directly.

Together with N, phosphorus (P) is also an important nutrient required for plant growth (Bergkemper et al., 2016). PGPR plays a key role in plant growth by facilitating the conversion of the available insoluble inorganic phosphate to the soluble PO${}_{4}^{3-}$ (Bergkemper et al., 2016). In PGPR, a mineral's phosphate-dissolving ability has been directly related to the presence of various genes responsible for producing organic acids. In this study, the genome of ASH15 contains genes coding for inorganic pyrophosphatase (*ppaC*) and alkaline phosphatase (*phoA*). The two-component system CS PhoB1/PhoR is involved in the alkaline phosphatase, phosphate starvation response (*phoH*), and an ABC transporter for phosphate uptake (*pstSCAB*), which are responsible for solubilizing the inorganic phosphate (Table 4). Moreover, the presence of an effective system in the PGPR for absorbing iron can help to protect the host plant from pathogen infestations (Herlihy et al., 2020; Lahlali et al., 2022). In the strain ASH15 genome, we also detected the presence of several siderophore-related genes in the ASH15 genome, including several iron ABC transporters (*fhuBD, afuABC*, and *FecCD)*, a ferric uptake regulator (*perR)*, an iron export ABC transporter permease *(fetB*), and a ferric transport system and ions import (*fhuBCG*) (Table 4). Our findings are in line with the fact that PGPR with salt-tolerant properties provides a range of benefits such as phytohormones, nitrogen fixation, P solubilization, ammonia production, and siderophore production for plant's stress tolerance and growth promotion (Egamberdieva et al., 2019; Arora et al., 2020; Khumairah et al., 2022).

In addition, genes like antimicrobial peptides and hydrolase genes, such as GTP cyclohydrolase (*ribBA*), α-amylase (*treC*), α-glucosidase (*malZ*), and glutamate dehydrogenase are also involved in plant immune responses. Moreover, oxidoreductase genes such as glutathione hydrolase proenzyme (ggt), superoxide dismutase (SOD), glutathione transport system (*gsiDCB*), and peroxiredoxin (DOT5, tpx) have been categorized. Strain ASH15′s genome predicted some key genes of volatile substances such as keto-acid metabolism (*ilvABCDEH*), 2,3-butanediol catabolism (*acuABC*), and Isopentenyl-diphosphate delta-isomerase (*idi*), which may be related to the biocontrol mechanism of strain ASH15.

### Biofilm-related genes in strain ASH15

The motility of bacteria is another important feature that enables them to move, colonize, and systematically spread in plants (Palma et al., 2022). The motility ability of strain ASH15 allows it to move through the soil matrix and into the plant, as confirmed by the genes involved in the flagella biosynthesis and assembly such as *flhA, flhB, flhF, flhG*; *fliAROPLJIHFTSW, flbD*; flagellar proteins *fliO/fliZ* and *flbD*; flagellar motor switch proteins, *fliN, fliY, fliM*, and *fliG*; and flagellar hook associated protein, *fliDEK* and *flgBCDEMN*, and two sets of genes coding for the flagellar motor proteins (*motA* and *motB*) (Table 4). Genome analysis of the strain ASH15 showed that two genes *hofN* and *pilBCAM* are involved in the biosynthesis and assembly of the type IV pilus system (T4PS): (Table 4).

### Secretion systems of strain ASH15

Bacteria have a set of different protein secretion systems that are essential for their growth and plant interaction. Bacteria secrete secondary metabolites, peptides, antibiotics, enzymes, and toxins to compete with nearby microbes or interact with host plants (Netzker et al., 2015; Köhl et al., 2019). Among the bacterial secretion systems, to transport proteins across the plasma membrane, the twin-arginine translocation (Tat) and general secretion (Sec) pathways are most commonly in use (Natale et al., 2008). The Tat pathway is mostly used to secrete folded proteins, while the Sec pathway primarily secretes unfolded proteins (Natale et al., 2008; Green and Mecsas, 2016). In this study, the strain ASH15 genome demonstrated five types of secretion systems: Type II/Type IV, Type III, Type VII (*yukDC*; ESX secretion system), and twin-arginine translocase (*tatAEC*). The operon *acuABC* encodes proteins to utilize acetoin and butanediol as carbon sources for energy requirements (Thanh et al., 2010). The presence of operon *acuABC* confirms that strain ASH15 has the ability to metabolize complex cyclic organic compounds and could be utilized for bioremediation.

### Genome mining for biosynthetic gene clusters and metabolic system analysis

**Figure 7:**
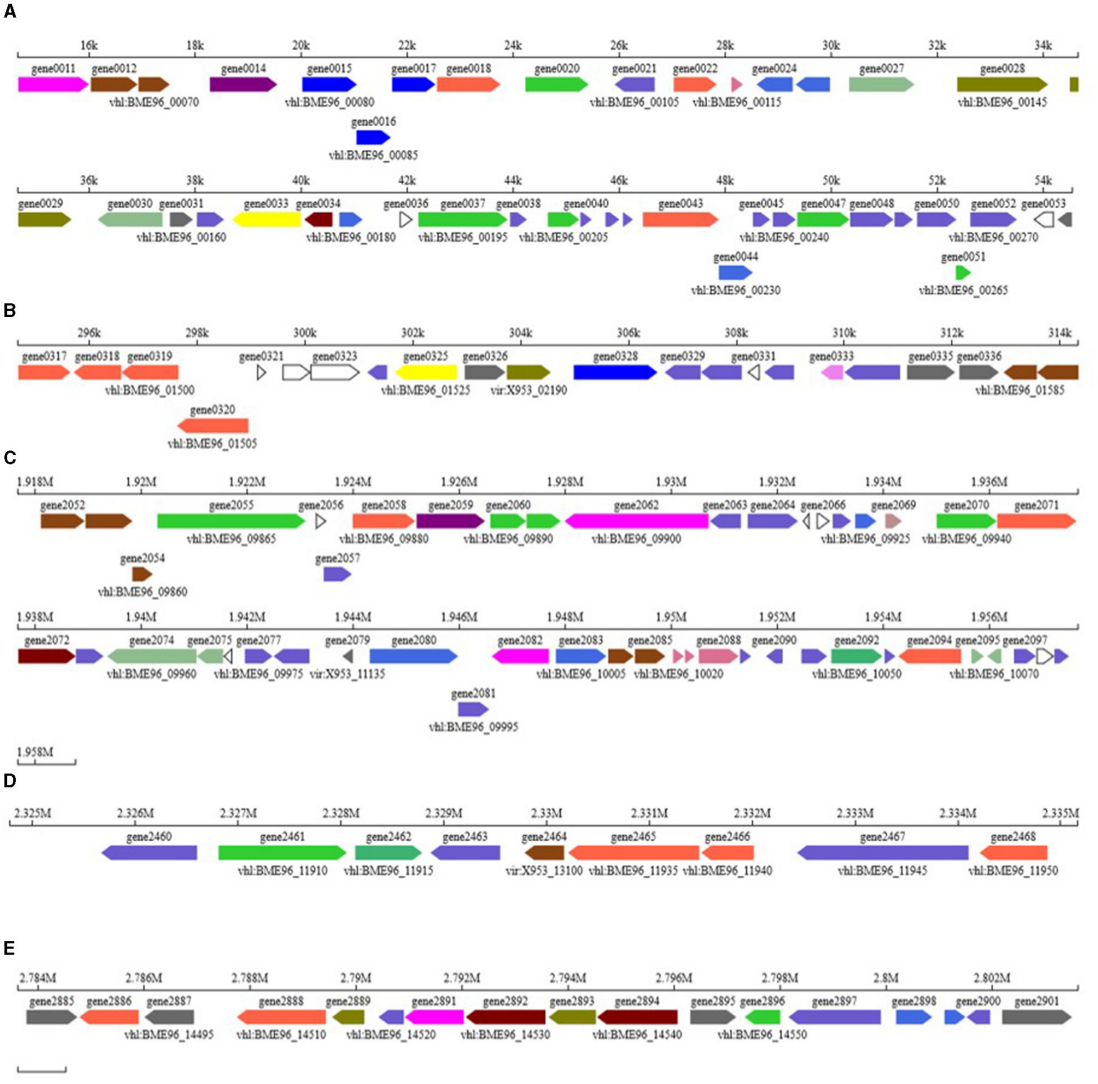
Biosynthesis gene clusters identified in the genome of *Virgibacillus halodenitrificans* strain ASH15. **(A)** T3PKS cluster with 45 genes; **(B)** terpene cluster with 23 genes **(C)** T3PKS cluster with 48 genes **(D)** ectoine cluster with 9 genes, and **(E)** terpene cluster with 17 genes.

The PGPR secretes bioactive secondary metabolites in the soil (rhizosphere-niche), which are related to plant-microbe interaction and root colonization as well as play a significant role in the plant immune response (Backer et al., 2018; Sharma et al., 2019; Bukhat et al., 2020; Jamali et al., 2020). The antimicrobial potential of strain ASH15 to produce hydrolytic enzymes and siderophores was confirmed by genome analysis (Tables 4, 5). The biosynthesis potential of the halophilic PGPR was assessed using antiSMASH 6.0.0 to predict both known and unidentified functional secondary metabolites in order to better understand its antagonistic action. Results showed (Figure 7) that five biosynthetic gene clusters were present in the genome: the first cluster of T3PKS (45 genes), the second cluster of terpenes (23 genes), the third cluster of T3PKS (48 genes), the fourth cluster of ectoine (9 genes), and the fifth cluster of terpenes (17 genes) (Figure 7).

**Table 5:** Sporulation/germination genes in *Virgibacillus halodenitrificans* strain ASH15.

**Characteristics**	**Gene IDs**	**Gene annotations**	**GO IDs**	**Chromosome location**
Sporulation/ germination	*yaaH*	Spore germination protein YaaH	GO:0005975	39995-38697
	*gerD*	Spore gernimation protein GerD	-	183600-182965
	*yndD*	Spore germination protein GerLA	GO:0009847	521045-522601
	*yndE*	Spore germination protein GerLB	GO:0009847	522603-523715
	*yndF*	Spore germination protein GerLC	GO:0009847	523804-524892
	*yfkR*	Spore germination protein GerQC	GO:0009847	841055-839922
	*GerAB*	GerAB/ArcD/ProY family transporter	GO:0009847	842167-841052
	*GerA*	Spore germination protein	GO:0009847	843668-842175
	*gerPF*	Spore germination protein PF	-	935193-934975
	*gerPE*	Spore germination protein PE	-	935623-935255
	*gerPD*	Spore germination protein PD	-	935809-935639
	*gerPC*	Spore germination protein PC	-	936391-935825
	*gerPB*	Spore germination protein PB	-	936670-936470
	*gerPA*	Spore germination protein PA	-	936897-936685
	*gerKA*	Spore germination protein KA	GO:0009847	1041166-1042710
	*-*	Spore germination protein YndE	-	1042694-1043785
	*-*	Spore germination protein GerQC	GO:0009847	1043782-1044918
	*-*	Spore germination protein XB	GO:0009847	1447960-1449033
	*-*	Spore germination protein XA	GO:0009847	1449030-1450457
	*-*	Spore germination protein XC	-	1450468-1451592
	*gerE*	Spore germination protein GerE	GO:0006355	1598214-1597990
	*gerM*	Spore germination protein GerM	-	1600644-1601720
	*gpr*	Germination protease	GO:0009847	1710456-1711565
	*spoVAF*	Spore germination protein	GO:0009847	1767148-1768605
	*spoIIAA*	Stage II sporulation protein AA	GO:0030435	1850841-1851194
	*spoIIAB*	Stage II sporulation protein AB	GO:0030435	1851191-1851631
	*sigH*	RNA polymerase sporulation-specific sigma factor	GO:0006352	1851640-1852392
	*spoVAA*	Stage V sporulation protein AA	GO:0016021	1852857-1853471
	*spoVAB*	Stage V sporulation protein AB	GO:0016021	1853452-1853877
	*spoVAF*	Stage V sporulation protein AF	GO:0009847	1853898-1855379
	*cwlJ*	Cell Wall Hydrolase	GO:0009847	1880202- 1881020
	*ypeB*	YpeB sporulation	GO:0009847	1881035-1882378
	*gerQ*	Spore coat protein GerQ	-	3432264-3432716
	*spoIIID*	Stage III sporulation protein D	GO:0003700	3352132-3351839
	*spoIIQ*	Stage II sporulation protein Q	GO:0016021	3355008-3354121
	*spoIID*	Stage II sporulation protein D	-	3357042-3355873
	*tasA*	Spore coat-associated protein	-	2562595-2562038
	*tasA*	Spore coat-associated protein	GO:0051301	2563259-2562663
	*tagT_U_V*	Polyisoprenyl-teichoic acid–peptidoglycan teichoic acid transferase	GO:0070726	2564384-2565328
	*tagT_U_V*	Polyisoprenyl-teichoic acid–peptidoglycan teichoic acid transferase	GO:0016021	2786947-2786009
	*ltaS*	Lipoteichoic acid synthase	GO:0016021	2584822-2582792
	*tagA*	WecB/TagA/CpsF family glycosyltransferase	GO:0071555	3280584-3279856
	*tagT_U_V*	Polyisoprenyl-teichoic acid–peptidoglycan teichoic acid transferase	GO:0016021	3280791-3281831
	*tagH*	Teichoic acids export ATP-binding protein TagH	GO:0005886	3293390-3292077
	*tagG*	Teichoic acid translocation permease protein TagG	GO:0055085	3294228-3293407
	*tagF*	Teichoic acid poly(glycerol phosphate) polymerase	-	3296463-3294349
	*inlB*	SH3-like domain-containing protein	-	3300922-3298235
	*lytD*	SH3-like domain-containing protein	-	3304409-3301056
	*tagD*	Glycerol-3-phosphate cytidylyltransferase	-	3304845-3305243
	*tagB*	CDP-glycerol glycerophosphotransferase family protein	-	3305236-3306417
	*tagD*	Glycerol-3-phosphate cytidylyltransferase	-	3306522-3306920
	*tagB*	CDP-glycerol glycerophosphotransferase family protein	-	3306910-3308055

Carbohydrates play several important roles in different biological functions. Carbohydrate activity enzymes derived from various species are divided into glycoside hydrolases (GHs), polysaccharide lyases (Ls), glycosyltransferases (GTs), auxiliary activities (AAs), carbohydrate-binding modules (CBMs), carbohydrate esterases (CEs), and other six major protein families. CAZyme analysis resulted in the identification of 80 genes in the genome ASH15 and annotated them (Figure 6, Supplementary Table 4) as 9 genes for AAs, 22 genes for CEs, 25 genes for GHs, and 24 genes for GTs.

### Other important genes identified in the genome

*V. halodenitrificans* strain ASH15 is an endospore-forming halophilic bacterium. Table 5 shows the predicted and identified genes (gene IDs, annotations, GO IDs, and chromosome locations) related to spore formation and spore germination in the genome of strain ASH15.

### Additional findings in the genome

Small RNAs play a significant role in metabolic pathway regulation and are thus very important. In the present investigation genome-wide analysis predicted a total of 120 sRNAs, which constitute 16,299 bases and 0.4252% of the strain ASH15 genome (Supplementary Table 2). We also identified tandem repeat sequences in the genome of strain ASH15. A total of 220 repeats were identified, which constitute 55,602 bases and 1.73% of the genome. Interspersed repeats, also known as transposable elements (transposon), includes DNA transposons and retrotransposons transposed by DNA-RNA. Common retrotransposons (also called Class I transposable elements or transposons via RNA intermediates) are LTR, LINE, and SINE. Genome analysis found that the strain has 6-SINE, 16-LINE, and 5 transposons, constituting 1,733 bases and 0.05% of the genome. Bacterial sRNA is a type of non-coding RNA with a length of 50 to 500 nt. They are mostly found in the intergenic region, while some are found in the coding genes' 5′ and 3′UTR sections. Bacterial sRNA mainly performs a variety of biological functions by binding to target mRNA or target protein. For example, bacterial sRNA plays an important role in regulating outer membrane protein expression, iron ion balance, community sense, and bacterial pathogenicity. Tandem repeats refer to the occurrence of two or more repeats in adjacent positions of the genome.

### Analysis of movable components of the genome

In the long evolutionary process, to adapt to changes in the environment or improve their own survival competitiveness, bacterial genomes often take in some foreign gene fragments and integrate them into their own genomes. These fragments generally contain some genes encoding specific functions, such as virulence genes, drug resistance genes, and metabolic genes, etc., which can change the phenotype of bacteria and help bacteria get through “difficulties” or occupy a dominant niche. These exogenous genome fragments are collectively called mobile elements; this phenomenon is called horizontal gene transfer (HGT). In the present genome analysis, three types of movable elements were predicted in strain ASH15: Genome Island, Prophage, and Clustered Regularly Interspaced Short Palindromic Repeats (CRISPR). Prophage is a bacteriophage genome integrated into the circular genome of the host bacteria (Piligrimova et al., 2021). As a ubiquitous mobile genetic element, prophage plays a key role in bacterial genetics, evaluation, and increasing survival and virulence potential through multiple mechanisms (Ramisetty and Sudhakari, 2019).

Conversely, the CRISPR system provides inherited and acquired sequence-specific adaptive immunity against the phage and other horizontally acquired elements like plasmid. CRISPR-associated (Cas) proteins constitute an RNA-guided adaptive immune system found in several prokaryotes. It is not only a type of bacteriophage defense system but also a regulator of bacterial physiology (Newsom et al., 2021). In recent times, to fulfill the demand for the next generation of industrial biotechnology (NGIB), the CRISPR/Cas system has been evaluated as a potential editing tool for customizing and reprogramming the genome of many extremophilic bacterial species for the production of natural compounds (Cress et al., 2016; Qin et al., 2018; Singh et al., 2021).

### Insertion sequences

The insertion sequence is a transposon encoding the enzyme required for transposition, and it is flanked by short, inverted terminal repeats. A total of 9 IS were identified in the genome of ASH15 using ISEScan software (Supplementary Figure 1).

### Genome island analysis

Genome island (GI) is one of the most important forms of horizontal transfer elements. It contains genes related to a variety of biological functions. According to the different genes, genome islands can generally be divided into virulence, drug-resistant, metabolic, symbiotic islands, among others. Genome islands are usually large, ranging from 10 to 200 kb. A total of 7 GIs were predicted in the genome, which constituted 162,069 bases, where the smallest island was 8,201 bases and the largest was 82,517 bases. The island genome has coded for 167 CDS, mainly belonging to proton transporters, iron transport, sugar transporters, chaperons, and many hypothetical proteins (Supplementary Figure 2 and Supplementary Table 3).

### Two-component systems identified in the genome of strain ASH15

Deep genome analysis revealed that in the strain ASH15 cell several two-component systems (nre*BC*, css*SR*, lia*SR*, pho*BR*, res*DE*, che*AY*, deg*US*, cit*TS*, yes*MN*, des*RKA*, wal*RK*, and vic*KR*) working, which play an important role in the modulation and regulation of the expression of critical proteins under stressful conditions. Specifically, nre*BC* is involved in nitrogen metabolisms, css*SR* is a sensory system, pho*BR* is associated with the phosphate metabolism mechanism, and histidine kinase sensor response regulators (res*DE*, yes*MN*, and desRK) are responsible for various cellular processes. Additionally, vicKR is a regulator for cell wall metabolism.

### LiaSR two-component system

Strain ASH15 has a *LiaIFRS* operon in the genome. The Lia*SR* is a two-component system widely found in Gram (+) bacteria. The Lia*SR* system is most studied in *Bacillus subtilis* as a part of the *LiaIHGFSR* operon. A striking characteristic of the Lia*SR* system is that its expression is induced upon exposure to antibiotics that target the cell envelope (Jordan et al., 2006; Suntharalingam et al., 2009; Shankar et al., 2015).

### CheAY, two-component system

CheAY two-component system plays a regulatory role in the signal transduction of chemotaxis (Zschiedrich et al., 2016). The CheY and CheA system are well studied in *B. subtilis* and *E. coli* (Rao et al., 2004; Minato et al., 2017). In *B. subtilis*, the autophosphorylating activity of CheA increases through the binding of attractants to transmembrane receptors (Karatan et al., 2001). Phosphorylated CheA donates the phosphate to the response regulator CheY (Wang et al., 2014). The CheY that has been phosphorylated interacts with the flagellar motor switch complex to insist the flagella rotate counterclockwise (CCW), which induces a smooth swimming motion (Minamino et al., 2019; Mukherjee et al., 2019).

### DegUS two-component system

DegUS two component system are identified in strain ASH15; it controls degradative enzyme synthesis. DegUS is involved in the complex network that mediates the regulation of transition state-specific processes. It contributes to controlling the development of natural competence for DNA uptake, motility, and degradative enzyme synthesis (Meliawati et al., 2022).

### CitST two-component system

The CitST system [carbon catabolite repression (CCR)] is involved in citrate fermentation metabolism and citrate/succinate transport. This two component system has also been studied in other bacterial genomes (Repizo et al., 2006).

### A unique finding in the genome of strain ASH15

#### Isoprenoid (squalene/phytoene) biosynthesis pathway

Genome analysis revealed that strain ASH15 has an MVA pathway to synthesize isoprenoids. The MVA pathway starts with the condensation of two acetyl-CoA molecules followed by a series of reduction (six) steps that produce IPP involving the expression (Figure 7) of genes: acs*A* (gene number 0346), HMG-CoA Synthase (gene number 00029; mva*S*), HMG-CoA reductase (gene number 0215; mvaE), mevalonate kinase (gene number 0216; mvaK1), phosphomevalonate kinase (gene number 0218; mva*K2*), and diphosphomevalonate decarboxylase (gene number 0217; mva*D*) (Figure 7). IPP and DMAPP are isomers, and inter-conversion is catalyzed by the enzyme isopentenyl-diphosphate Delta-isomerase (IdI). id*I* (gene number 0398) is the key regulatory gene that maintains the IPP to DMAPP ratio and has a significant impact on isoprenoid biosynthesis. The gene (gene number 1929), which codes for geranylgeranyl diphosphate synthase (GGPPS), converts farnesyl diphosphate (FPP) to geranylgeranyl diphosphate (GGPP). Farnesyl diphosphate (FPP) is an intermediate molecule that could be converted to many different terpenoids (Rinaldi et al., 2022), while GGPP could be further converted to diterpenoids and tetraterpenoids. Strain ASH15 has genes (SQS_PSY; 0327 and SQS_PSY; 2893) to produce triterpenoids and tetraterpenoids, Squalene (C30) and Phytoene (C40). Squalene synthase (gene number 0327) converts two molecules of FPP to squalene, and phytoene synthase (gene number 2893) could convert GGPP to Phytoene.

Squalene has been known for its various applications, such as an anti-cancer agent, an anti-oxidant agent, an anti-bacterial agent, a chemopreventive agent, an anti-aging agent, a detoxifier, and an adjuvant for drug carriers and vaccines (Kim and Karadeniz, 2012; Paramasivan and Mutturi, 2022). Therefore, it has enormous potential in the food, cosmetics, and pharmaceutical industries (Huang et al., 2009; Gohil et al., 2019). The global squalene market demand in 2014 was approximately 2.67 kilotons, and by 2022, it is estimated to reach a value of USD$ 241.9 million, with the majority of sales coming from personal care and cosmetic items (Rosales-Garcia et al., 2017). There is a pressing need to generate squalene in a renewable and sustainable way to meet this ever-increasing demand. The development of microbial cell factories could be a solution to fulfill this demand, and strain ASH15 is a natural bacterium that has all the genes for the biosynthesis of squalene.

## Conclusion

According to the findings of the present study, the availability of *V. halodenitrificans* ASH15's entire genome will shed additional light on complex biological systems, which showed that strain ASH15 has open a number of opportunities to study this efficient plant growth-promoting bacterium. These results indicate that strain ASH15 may be used as a possible eco-friendly bioresource alternative for chemical fertilizers to promote plant growth in salt stressed agriculture area. However, the usability of *V. halodenitrificans* ASH15 under field trials is required for establishing it as a potential plant growth promoter for utilizing in sustainable agriculture under saline conditions.

## Data availability statement

The datasets presented in this study can be found in online repositories. The names of the repository/repositories and accession number(s) can be found below: https://www.ncbi.nlm.nih.gov/nuccore/CP090006.1/.

## Author contributions

AS, X-PS, and Y-RL conceived the idea and designed the experiments. AS performed the experiments and wrote the original draft of the manuscript. RNS assisted in analysis and software support. RS, PS, and D-JG assisted in the experiments. X-PS contributed to resource management. KV and Y-RL critically revised the manuscript. All authors have read and agreed to the published version of the manuscript.
