# Supplementary material for: Genome analysis of a halophilic Virgibacillus halodenitrificans ASH15 revealed salt adaptation, plant growth promotion, and isoprenoid biosynthetic machinery
Source: Front Microbiol. 2023 Sep 22;14:1229955. doi: 10.3389/fmicb.2023.1229955 (PMC10556750; doi:10.3389/fmicb.2023.1229955)
Supplement: Supplementary file 1 [file Data_Sheet_1.pdf]

**Supplementary Figure SF-1. A-I (IS1-IS9) Insertion Sequences identified the in the genome of strain ASH15**

A:IS1

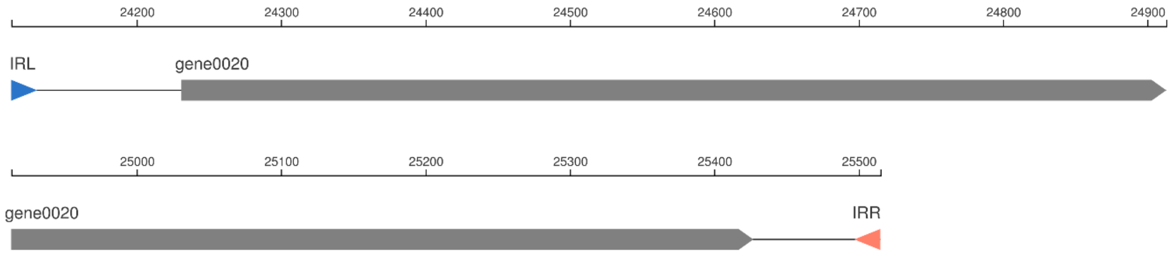


B:IS2

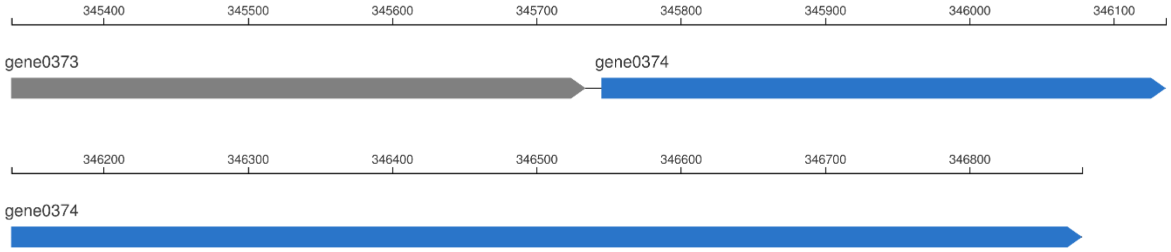


C:IS3

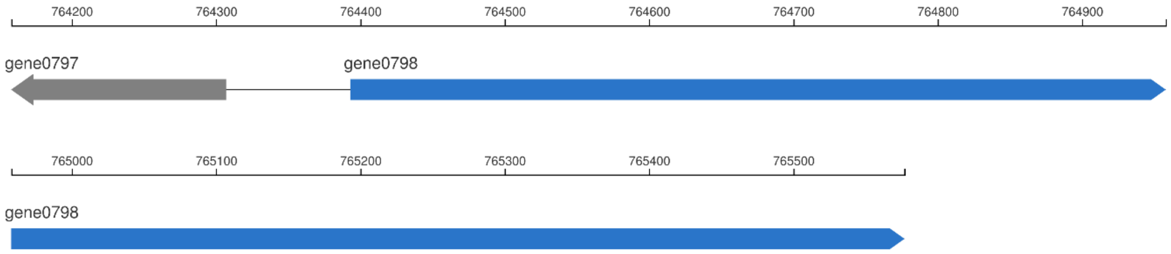


D:IS4

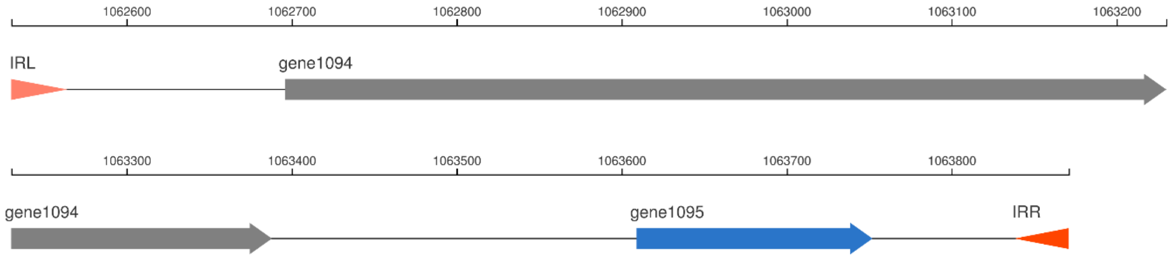


E:IS5

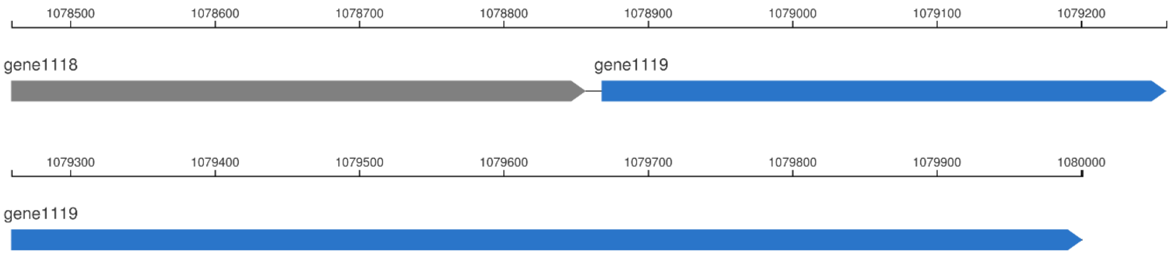


F:IS6

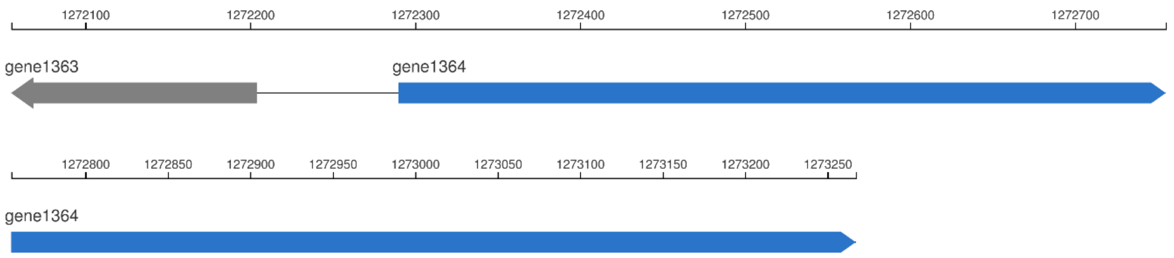


G:IS7

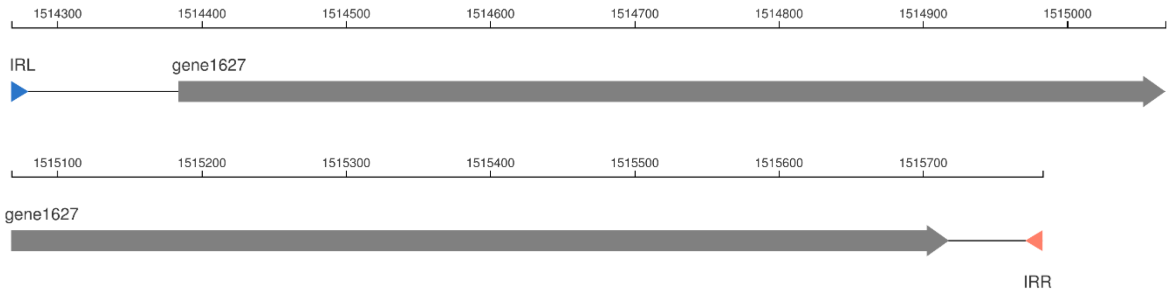


H:IS8

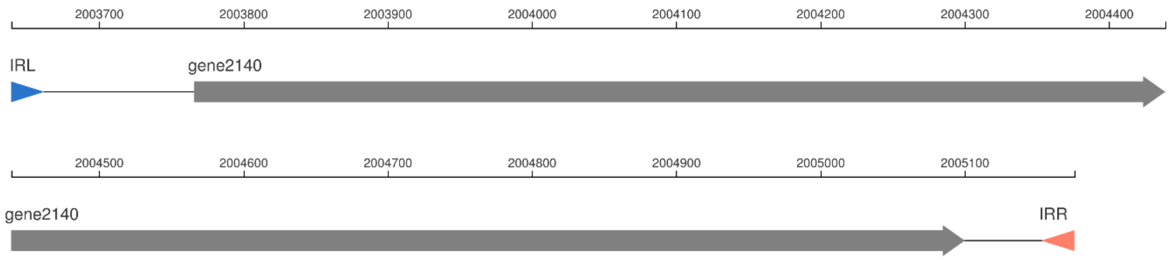


I:IS9

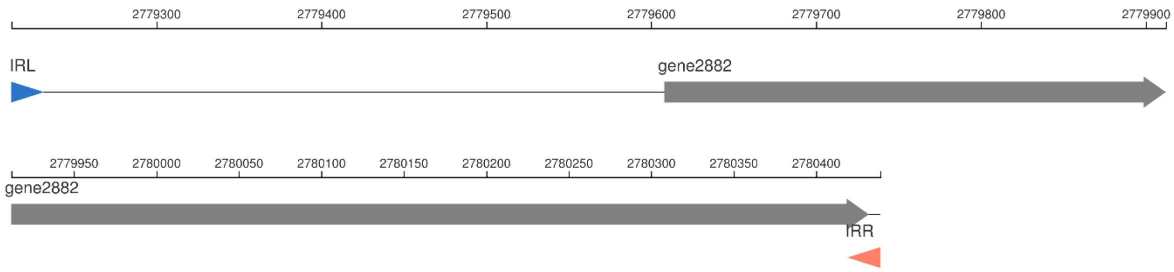

**Supplementary Figure SF-2: Figure S5: A-G (GI01-GI07) Genomic islands identified in the genome ASH15**

A:GI01

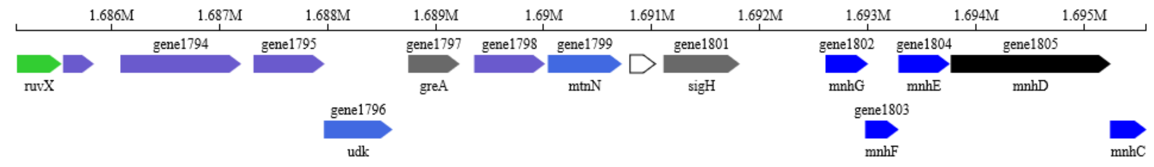


B:GI02

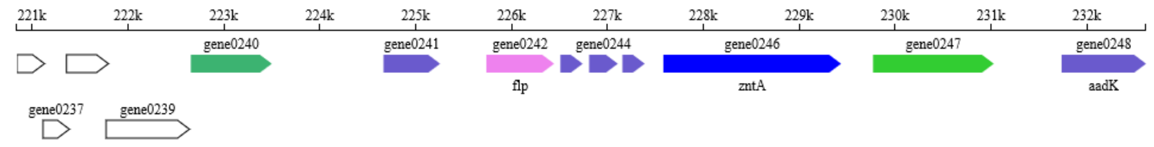


C:GI03

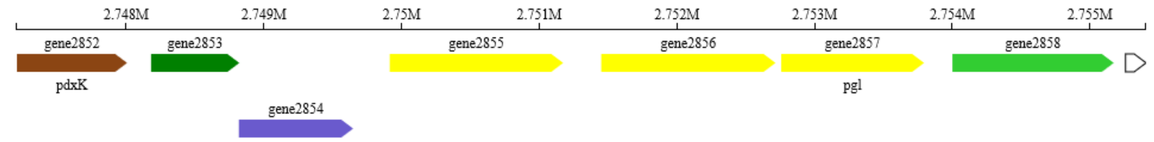


D:GI04

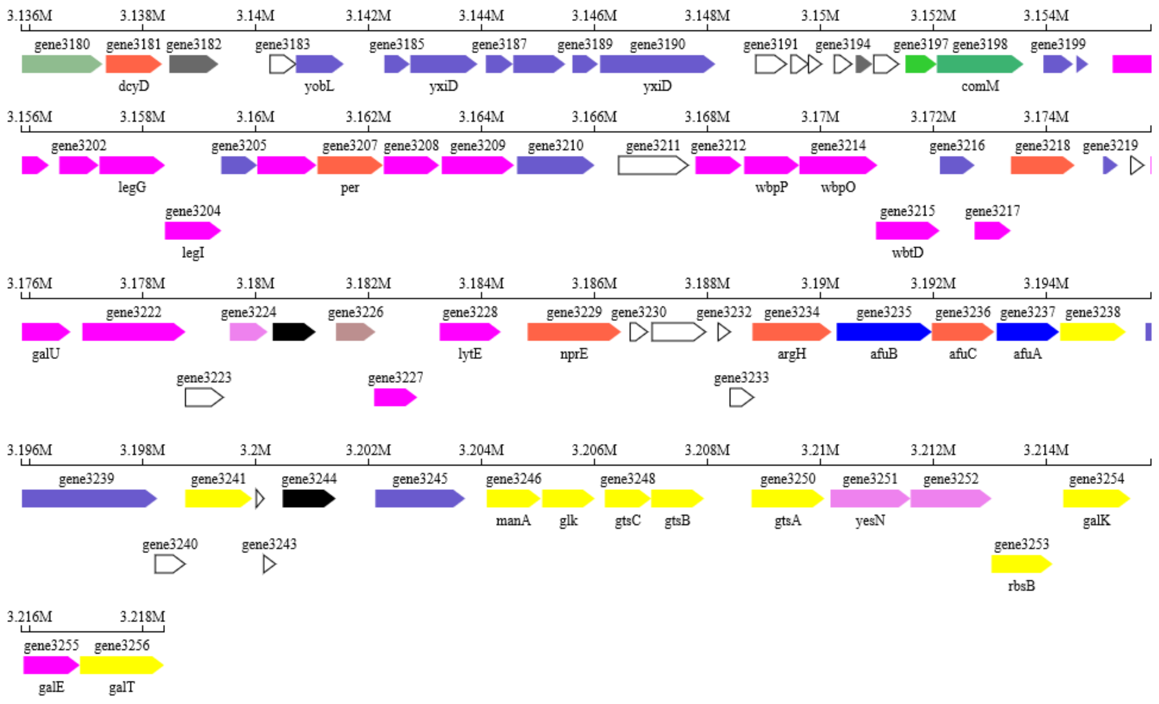

E:GI05

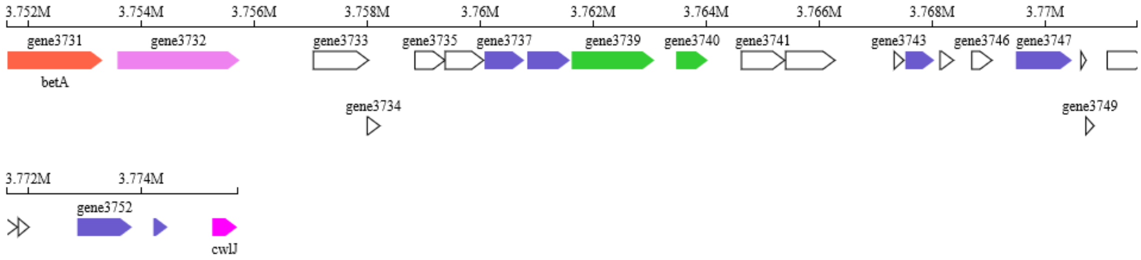


F:GI06

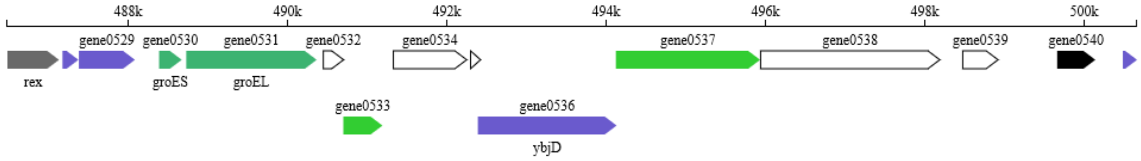


G:GI07

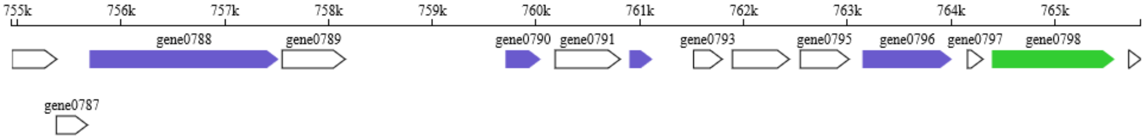

**Supplementary Figure SF-3: CRISPR-Cas analysis.**

CRISPRs identified in *Virgibacillus*

CRISPR1

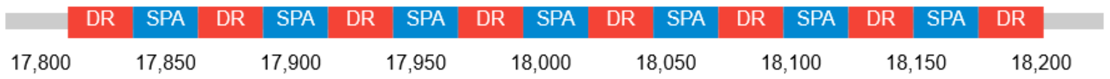


CRISPR2

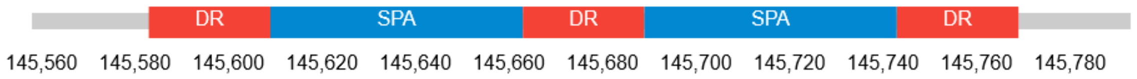


CRISPR3

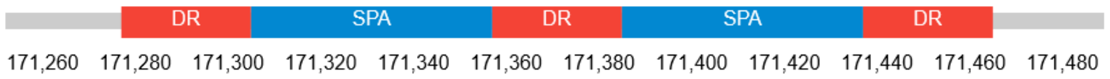


CRISPR4

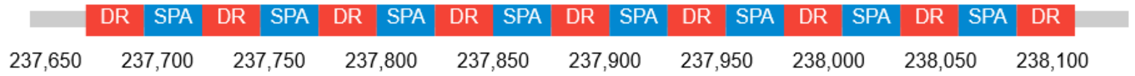


CRISPR5

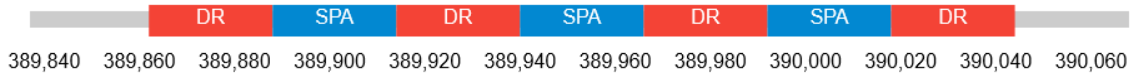


CRISPR6

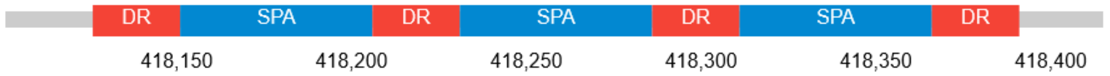


CRISPR7

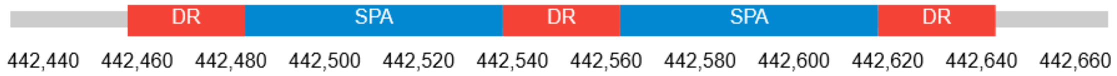


CRISPR8

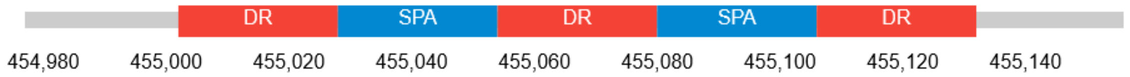


CRISPR9

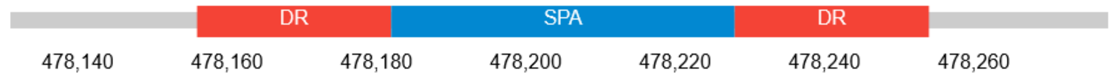


CRISPR10

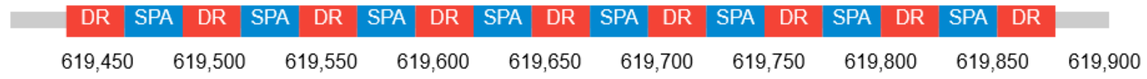


CRISPR11

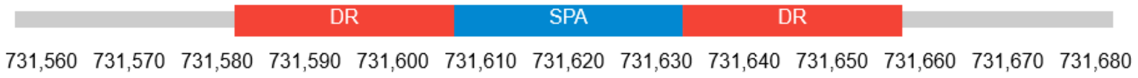


CRISPR12

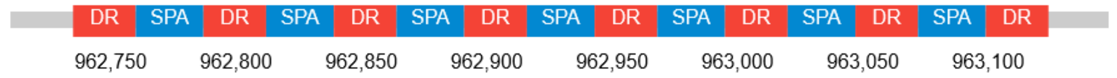


CRISPR13

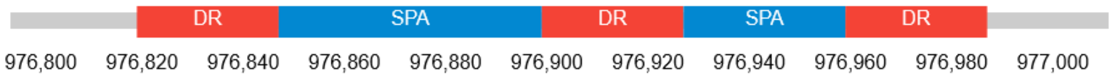


CRISPR14

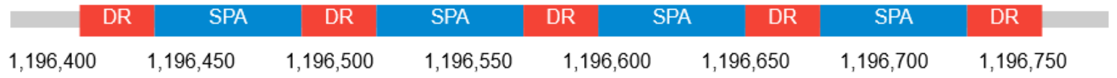


CRISPR15

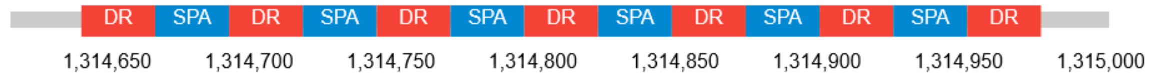


CRISPR16

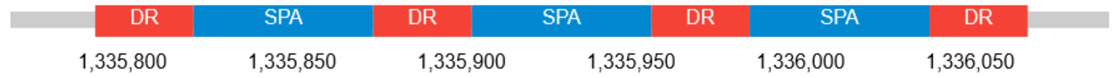


CRISPR17

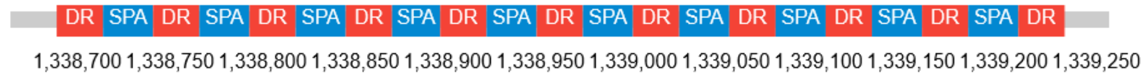


CRISPR18

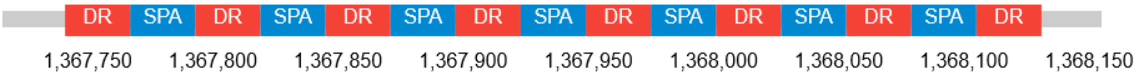


CRISPR19

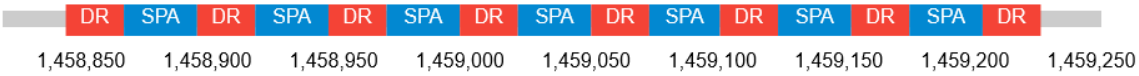


CRISPR20

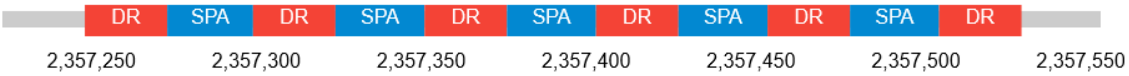


CRISPR21

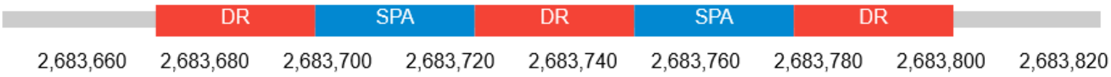


CRISPR22

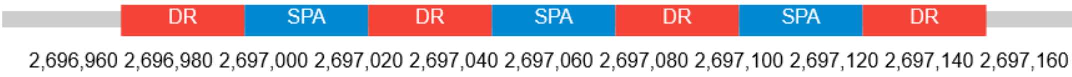


CRISPR23

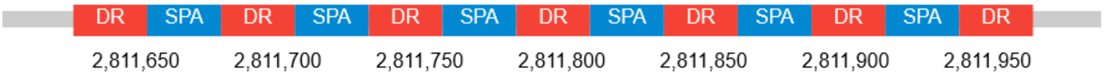


CRISPR24

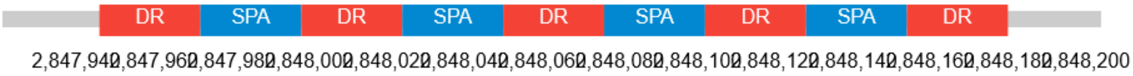


CRISPR25

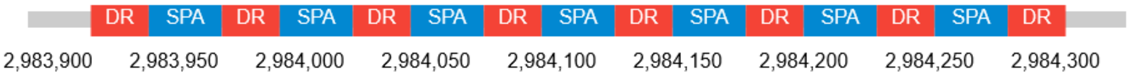


CRISPR26

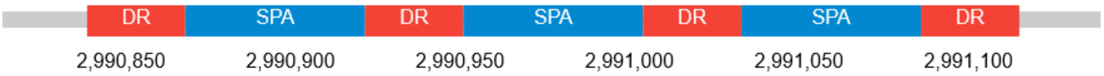


CRISPR27

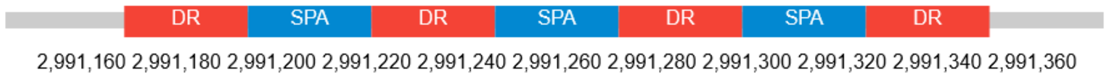


CRISPR28

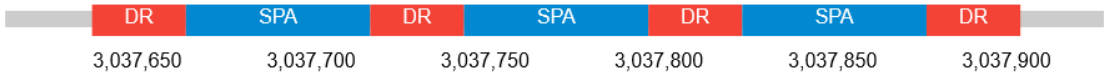


CRISPR29

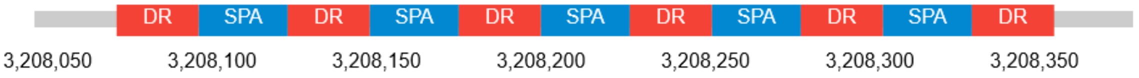


CRISPR30

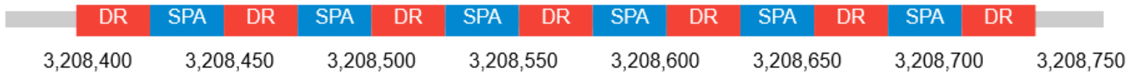


CRISPR31

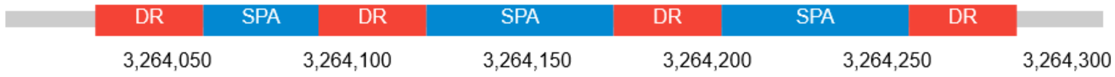


CRISPR32

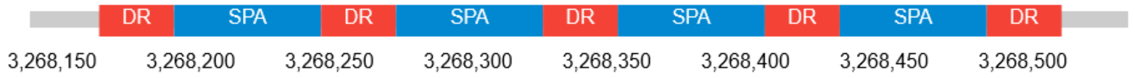


CRISPR33

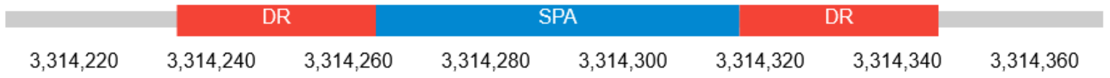


CRISPR34

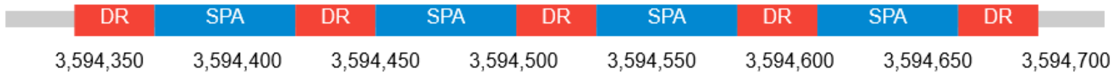


CRISPR35

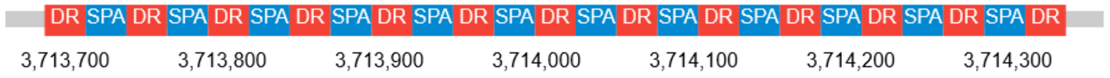


CRISPR36

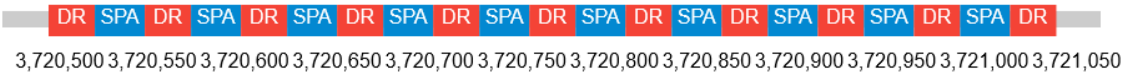
DR SPA DR SPA DR SPA DR SPA DR SPA DR SPA DR SPA DR SPA DR SPA DR SPA DR SPA DR
3,720,500 3,720,550 3,720,600 3,720,650 3,720,700 3,720,750 3,720,800 3,720,850 3,720,900 3,720,950 3,721,000 3,721,050

## Supplementary Table 1. Carbon source utilization by the strain ASH15

| S. No. | Serial No. (Biolog) | Substrate | Chemical Guild | ASH15 | S. No. | Serial No. (Biolog) | Substrate | Chemical Guild | ASH15 |
|---|---|---|---|---|---|---|---|---|---|
| 1 | A1 | Negative Control | Sugars | - | 25 | C1 | α-D-Glucose | Sugars | + |
| 2 | A2 | Dextrin | Sugars | - | 26 | C2 | D-Mannose | Sugars | + |
| 3 | A3 | D-Maltose | Sugars | + | 27 | C3 | D-Fructose | Sugars | + |
| 4 | A4 | D-Trehalose | Sugars | + | 28 | C4 | D-Galactose | Sugars | + |
| 5 | A5 | D-Cellobiose | Sugars | + | 29 | C5 | 3-Methyl Glucose | Sugars | + |
| 6 | A6 | Gentiobiose | Sugars | + | 30 | C6 | D-Fucose | Sugars | + |
| 7 | A7 | Sucrose | Sugars | + | 31 | C7 | L-Fucose | Sugars | + |
| 8 | A8 | D-Turanose | Sugars | + | 32 | C8 | L-Rhamnose | Sugars | + |
| 9 | A9 | Stachyose | Sugars | + | 33 | C9 | Inosine | Sugars | + |
| 10 | A10 | Positive Control | Chemical Sensivity | + | 34 | C10 | 1% Sodium Lactate | Lactic acid | - |
| 11 | A11 | pH 6 | Acidic pH | + | 35 | C11 | Fusidic Acid | Chemical Sensivity | + |
| 12 | A12 | pH 5 | Acidic pH | - | 36 | C12 | D-Serine | Chemical Sensivity | + |
| 13 | B1 | D-Raffinose | Sugars | - | 37 | D1 | D-Sorbitol | Chemical Sensivity | - |
| 14 | B2 | α-D-Lactose | Sugars | + | 38 | D2 | D-Mannitol | Chemical Sensivity | + |
| 15 | B3 | D-Melibiose | Sugars | + | 39 | D3 | D-Arabitol | Chemical Sensivity | + |
| 16 | B4 | β-Methyl-D-Glucoside | Sugars | + | 40 | D4 | myo-Inositol | Chemical Sensivity | + |
| 17 | B5 | D-Salicin | Sugars | + | 41 | D5 | Glycerol | Chemical Sensivity | + |
| 18 | B6 | N-Acetyl-D-Glucosamine | Sugars | + | 42 | D6 | D-Glucose-6-$PO_4$ | Hexose-$PO_4$ | + |
| 19 | B7 | N-Acetyl-β-D Mannosamine | Sugars | + | 43 | D7 | D-Fructose-6-$PO_4$ | Hexose-$PO_4$ | + |
| 20 | B8 | N-Acetyl-D-Galactosamine | Sugars | + | 44 | D8 | D-Aspartic Acid | Chemical Sensivity | + |
| 21 | B9 | N-Acety l Neuraminic Acid | Sugars | + | 45 | D9 | D-Serine | Chemical Sensivity | + |
| 22 | B10 | 1% NaCl | Sodium Chloride | + | 46 | D10 | Troleandomycin | Chemical Sensivity | + |
| 23 | B11 | 4% NaCl | Sodium Chloride | + | 47 | D11 | Rifamycin SV | Chemical Sensivity | + |
| 24 | B12 | 8% NaCl | Sodium Chloride | + | 48 | D12 | Minocycline | Chemical Sensivity | - |

## Continue supplementary Table 1.

| S. No. | Serial No. (Biolog) | Substrate | Chemical Guild | ASH15 | S. No. | Serial No. (Biolog) | Substrate | Chemical Guild | ASH15 |
|---|---|---|---|---|---|---|---|---|---|
| 49 | E1 | Gelatin | Amino acid | - | 73 | G1 | p-Hydroxy- Phenylacetic Acid | Carboxylic acids, esters and fatty acids | - |
| 50 | E2 | Glycyl-L-Proline | Amino acid | + | 74 | G2 | Methyl Pyruvate | Carboxylic acids, esters and fatty acids | + |
| 51 | E3 | L-Alanine | Amino acid | + | 75 | G3 | D-Lactic Acid Methyl Ester | Carboxylic acids, esters and fatty acids | + |
| 52 | E4 | L-Arginine | Amino acid | + | 76 | G4 | L-Lactic Acid | Carboxylic acids, esters and fatty acids | + |
| 53 | E5 | L-Aspartic Acid | Amino acid | + | 77 | G5 | Citric Acid | Carboxylic acids, esters and fatty acids | - |
| 54 | E6 | L-Glutamic Acid | Amino acid | + | 78 | G6 | α-Keto-Glutaric Acid | Carboxylic acids, esters and fatty acids | - |
| 55 | E7 | L-Histidine | Amino acid | + | 79 | G7 | D-Malic Acid | Carboxylic acids, esters and fatty acids | - |
| 56 | E8 | L-Pyroglutamic Acid | Amino acid | + | 80 | G8 | L-Malic Acid | Carboxylic acids, esters and fatty acids | + |
| 57 | E9 | L-Serine | Amino acid | + | 81 | G9 | Bromo-Succinic Acid | Carboxylic acids, esters and fatty acids | + |
| 58 | E10 | Lincomycin | Chemical Sensivity | + | 82 | G10 | Nalidixic Acid | Chemical Sensivity | + |
| 59 | E11 | Guanidine HCl | Chemical Sensivity | + | 83 | G11 | Lithium Chloride | Chemical Sensivity | - |
| 60 | E12 | Niaproof 4 | Chemical Sensivity | - | 84 | G12 | Potassium Tellurite | Chemical Sensivity | - |
| 61 | F1 | Pectin | Hexose acid | - | 85 | H1 | Tween 40 | Carboxylic acids, esters and fatty acids | - |
| 62 | F2 | D-Galacturonic Acid | Hexose acid | + | 86 | H2 | γ-Amino-Butryric Acid | Carboxylic acids, esters and fatty acids | + |
| 63 | F3 | L-Galactonic Acid Lactone | Hexose acid | + | 87 | H3 | α-Hydroxy- Butyric Acid | Carboxylic acids, esters and fatty acids | + |
| 64 | F4 | D-Gluconic Acid | Hexose acid | + | 88 | H4 | β-Hydroxy-D, L Butyric Acid | Carboxylic acids, esters and fatty acids | + |
| 65 | F5 | D-Glucuronic Acid | Hexose acid | + | 89 | H5 | α-Keto-Butyric Acid | Carboxylic acids, esters and fatty acids | + |
| 66 | F6 | Glucuronamide | Hexose acid | + | 90 | H6 | Acetoacetic Acid | Carboxylic acids, esters and fatty acids | + |
| 67 | F7 | Mucic Acid | Hexose acid | + | 91 | H7 | Propionic Acid | Carboxylic acids, esters and fatty acids | + |
| 68 | F8 | Quinic Acid | Hexose acid | + | 92 | H8 | Acetic Acid | Carboxylic acids, esters and fatty acids | + |
| 69 | F9 | D-Saccharic Acid | Hexose acid | + | 93 | H9 | Formic Acid | Carboxylic acids, esters and fatty acids | + |
| 70 | F10 | Vancomycin | Chemical Sensivity | + | 94 | H10 | Aztreonam | Chemical Sensivity | + |
| 71 | F11 | Tetrazolium Violet | Reducing Sugar | - | 95 | H11 | Sodium Butyrate | Chemical Sensivity | - |
| 72 | F12 | Tetrazolium Blue | Reducing Sugar | - | 96 | H12 | Sodium Bromate | Chemical Sensivity | - |

‘+’ positive for utilization; ‘-‘ negative for utilization.
